# Genotype to Phenotype Maps: Multiple Input Abiotic Signals Combine to Produce Growth Effects via Attenuating Signaling Interactions in Maize

**DOI:** 10.1534/g3.113.008573

**Published:** 2013-10-18

**Authors:** G. Buddhika Makumburage, H. Lee Richbourg, Kalindi D. LaTorre, Andrew Capps, Cuixen Chen, Ann E. Stapleton

**Affiliations:** * Department of Mathematics and Statistics, University of North Carolina Wilmington, Wilmington, North Carolina 28401; † Department of Biology and Marine Biology, University of North Carolina Wilmington, Wilmington, North Carolina 28401

**Keywords:** abiotic stress, multiple stress, recombinant inbred line, nested association mapping, modifier networks

## Abstract

The complexity of allele interactions constrains crop improvement and the prediction of disease susceptibility. Additive allele effects are the foundation for selection in animal and plant breeding, and complex genetic and environmental interactions contribute to inefficient detection of desirable loci. Manipulation and modeling of other sources of variation, such as environmental variables, have the potential to improve our prediction of phenotype from genotype. As an example of our approach to analysis of the network linking environmental input to alleles, we mapped the genetic architecture of single and combined abiotic stress responses in two maize mapping populations and compared the observed genetic architecture patterns to simple theoretical predictions. Comparisons of single and combined stress effects on growth and biomass traits exhibit patterns of allele effects that suggest attenuating interactions among physiological signaling steps in drought and ultraviolet radiation stress responses. The presence of attenuating interactions implies that shared QTL found in sets of environments could be used to group environment types and identify underlying environmental similarities, and that patterns of stress-dependent genetic architecture should be studied as a way to prioritize prebreeding populations. A better understanding of whole-plant interactor pathways and genetic architecture of multiple-input environmental signaling has the potential to improve the prediction of genomic value in plant breeding and crop modeling.

Mechanistic understanding and prediction of the relationship between genotype and phenotype depend on the genetic architecture—the number of large-effect and small-effect genes and the way these alleles interact to generate variation in traits. At the molecular scale, nonlinear epistatic interactions between different genes are common, whereas at the population and evolutionary scales, additive infinitesimal models of genetic architecture are widespread and successful in explaining observed patterns (Lynch and Walsh 1998; Hill *et al.* 2008). The genetic architecture is plastic, with causal alleles in one context typically becoming unimportant in different environments (Lynch and Walsh 1998; Crossa 2012; Tardieu 2012). This environmental input dependency has been modeled by Cooper and Podlich (2002) as E(NK), with environment (E) affecting genes (N) and gene interactions (K). In this formulation, increasing the number of modifying environments deforms the parameter space, leading to local optima and saddles between optima. This nonuniform parameter space for genotype–environment interaction was seen in a comprehensive analysis of genetic architecture in barley, in which increasing the number of environments up to 22 decreased the number of general environmental-controlling loci and increased the number of environment subset-specific loci (Lacaze *et al.* 2008).

Representation of allele interactions as edges in a network is often used in molecular-scale models of gene regulation and provides a useful abstraction when considering alternative models of genotype–environment interaction. Recent work on control of networks indicates that relatively large numbers of separately controlled genes are needed to specify gene regulation output state (Liu *et al.* 2011), which fits with the formulation of E modifying NK at the whole-plant level. Liu *et al.* (2011) also found that densely interconnected networks rapidly decrease the number of nodes needed to specify the output state, which is also consistent with the finding that modeling of crop-scale output landscapes generates complex cross-sections and local optima (Cooper and Podlich 2002). Signaling networks regulate multiple downstream activities in cells and tissues, in contrast to ongoing metabolic homeostatic processes (Trevino Santa Cruz *et al.* 2005). Signaling implies input control, analogous to E controlling NK or input signaling to driver nodes in networks. At the highest level of aggregation, signal networks can be mapped to logical operators to predict output (Trevino Santa Cruz *et al.* 2005; Mittenthal and Zou 2011). Signals at this higher level of abstraction may encapsulate or control physiological effects at the whole-organism level (Martin *et al.* 2011). Models of this top hierarchical level thus focus on prediction of combined inputs on the same output (Trevino Santa Cruz *et al.* 2005).

Although environment-specific alleles are commonly identified and are often ascribed to additional (unspecified) environmental inputs, controlled combined stress experiments are rare (Mittler 2006). Phenotypic data regarding the combination of ultraviolet (UV) radiation and drought, however, are available for several species. For example, a protective response to combined stress is observed in soybean, in which factorial experiments with increased UV-B and drought resulted in growth inhibition similar to the level seen in the individual stress treatments (Murali and Teramura 1986; Sullivan and Teramura 1988). This protective response suggested that photomorphogenic changes such as stomatal density alterations might confer drought tolerance (Gitz and Liu-Gitz 2003). Factorial experiments measuring drought and UV radiation in wheat suggested that increases in lipid peroxidation products generated a protective response in the combination drought plus UV-B treatment group (Alexieva *et al.* 2001).

In these soybean and wheat cases, the two input stresses applied in combination were not additive. Genetic differences were not examined in either study, however. The nonlinear effect of UV and drought in these cases motivated us to examine the genetic architecture of these particular stresses in maize mapping populations. Maize is a model system and crop of major economic importance; corn acreage in the United States covers approximately 32 million hectares per year (Baker 2010). The most important abiotic stress in maize is drought; limited water availability reduces yield worldwide (Boyer 1982). Projections of crop effects under climate change indicate that water availability will decline and water demand will increase (Schneider *et al.* 2007). Additional abiotic stress factors important for maize yield include nitrogen insufficiency, high or low temperatures, UV radiation increases, mineral deficiency or toxicity, and ozone (Gao *et al.* 2004; Collins *et al.* 2008).

We examined the role of environmental inputs in an experimental test of abiotic factor interactions by comparison of patterns of genetic architecture. We used the tractable and publiclly available maize (*Zea mays* L.) mapping population resources to identify loci with significant genotype–environment interactions for plant growth traits. These mapping resources have complementary strengths, with the maize intermated recombinant inbred populations providing high resolution via relatively small introgressed regions (Lee *et al.* 2002) and the nested association mapping population providing allelic comparisons to a reference allele across the subpopulations (Yu *et al.* 2008). Factorial environmental experiments, in contrast to analyses that fit environmental variation from weather and soil records, require controlled conditions and are thus limited by the availability of managed stress environments rather than field records. Thus, analysis of multiple mapping populations for trends in genetic architecture is more tractable than very large factorial experiments with one mapping population—and multiple populations address genetic generality rather than focusing on high power to detect allele and population-specific patterns. For our analysis of factorial environmental inputs in these two maize mapping populations, we defined three simple logical expectations for the genetic architecture of combined stress inputs using the Trevino Santa Cruz *et al.* (2005) formulation for our expected maize stress effects (Figure 1). Our three possible combinations of two inputs are independent effect (Figure 1A), an AND gate (Figure 1B), and an OR gate (Figure 1C). Each of these combination types generates a prediction for the genetic architecture in quantitative trait locus (QTL)/association mapping experiments. If signal transmission for each stress is indeed independent, then alleles or genes important for differences in growth under drought would be different from the alleles important for growth under UV radiation (Figure 1A). Different genes are by definition at different genomic locations, so mapping of the location of the important allelic differences (single nucleotide polymorphism or marker state) for independent inputs would result in identification of different nonoverlapping sets of loci for the two stresses. In the combined stress environment, the union of these sets would be predicted. Typical-size QTL mapping experiments such as ours have a detection limit of tens of loci (Lynch and Walsh 1998). This means that the prediction for combined stress is for truncated lists instead of the complete union; the effect size of the important alleles (in other words, the rank order in the list) is thus also needed for interpretation of the expected effect in the combined stress. For the independence case, we expect that the loci would show consistent effects—for example, the UV locus with the largest effect should also appear on the combined stress important locus list. Nonindependent stress signaling could result from specific signal detection (an AND gate), as diagrammed in Figure 1B, or from shared signal input integration (Figure 1C). The genetic architecture for combination stress-specific signaling (AND gate) (Figure 1B) would show novel loci for the two-stress experimental treatment, with the detection limit on the total number of loci determining how many single stress-specific loci are still visible in the joint stress treatment. A third alternative is stress input combination signaling through integrators (OR gates) (Figure 1C), with the integrator function responsive to signals from either stress. Some integrators for plant stress signaling have been molecularly identified, such as reactive oxygen species and hormones (Tognetti *et al.* 2012). The genetic architecture prediction for OR gate integrator alleles is that the same loci will be identified in both stresses and in the combined stress (fully overlapping sets).

**Figure 1:**
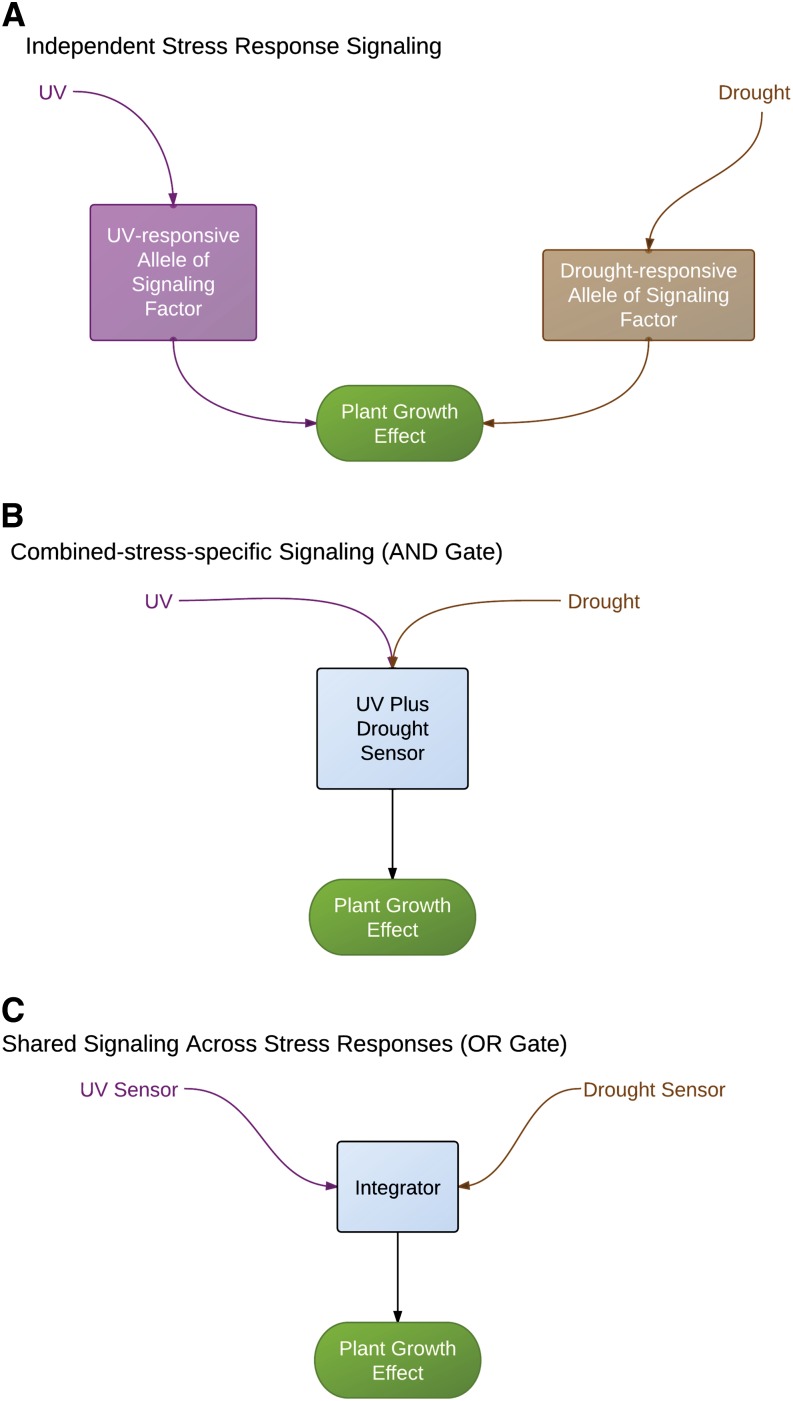
Alternative pathways for two-stress signaling. Square boxes indicate signaling or perception steps with genetic variation that could be detected as QTL. (A) Two abiotic stress treatments are perceived by separate signaling pathways. The effect of the combined stress is predictable by adding or multiplying the individual allele effects. (B) The two abiotic stress treatments are perceived by a sensor that is only activated by those two stress signals applied together (an AND gate). (C) The two abiotic stress treatments are perceived by a single integrative cellular component (*e.g.*, a transcription factor or reactive oxygen species sensor), with the effect of each stress increasing the output signal (an OR gate). The effect of a combined stress at a locus encoding an integrator is assumed to be predictable from the single-stress allelic effects.

We tested our simplified predictions (Figure 1) for response to a combination of two abiotic stresses in biparental and multiparental maize mapping populations for three traits. The presence of an allele with a large effect in one stress was not a good predictor of the importance of that allele in a combined stress environment. We propose a signaling circuitry with repressive attenuating modifier loci that better explains our experimentally observed genetic architecture.

## Materials and Methods

### Mapping populations

Traditional recombinant inbred line (RIL) QTL mapping experiments contain alleles from two parents, with intermating in early generations providing increased resolution in populations such as the maize intermated B73-Mo17 (IBM) set (Lee *et al.* 2002) that we used for mapping. Current large-scale production of RIL resources (www.panzea.org) provides the opportunity to consider effects of alleles contributed by multiple parents (Holland 2007). The common parent in the maize nested association mapping (NAM) recombinant inbred line resource is B73; this inbred line is the source of the public maize genome sequence data (Schnable *et al.* 2009). However, the NAM population was not intermated, and the nested contributing populations confer trade-offs in choosing populations subsets for more intensive analyses (Yu *et al.* 2008). We used the first 50 recombinant inbred lines from five different parental populations, Z005[non-B73 parent CML277], Z010[Hp301], Z011[IL14H], Z016[M37W], and Z022[Oh43].

### Abiotic stress treatments and trait measurements

The IBM94 maize populations were grown in the Kresge greenhouse located on the University of North Carolina Wilmington campus. Each replicate of the experiment consisted of four treatment groups: control; drought; UV; and UV plus drought. Experiment 1 was conducted in May, experiment 2 was conducted in June, experiment 3 was conducted in July, and experiment 4 was conducted in August of 2007 (n = 4). By replicating across time we increased the generality of our results and optimized the power to detect genotype–environment interactions for the drought and UV radiation factors within each block. Ninety-two RILs from the IBM94 population were evaluated in this series of experiments. In addition, check plants were added for comparison across the 4-month blocks. Plants were grown in 11.-4cm plastic pots for 10 d before beginning experimental treatment. Plants were irrigated every other day from sowing through the eighth day after planting. The full water weight was measured for pots containing soil and no plants, and plant height was recorded for each plant before stress. Plants in the drought and UV plus drought combined treatment groups were not watered again until the 3-d treatment was over, whereas plants in the control and UV treatment groups continued to be watered each day. Pot weights were recorded again on the first day after treatment to gauge the drought intensity on the drought treatment groups relative to the control group. Final height measurements were recorded 7 d after treatment, for a total of 10 d between initial and final measurements. Dry weights of root tissue were collected after complete drying of root mass as judged by no further decrease in mass.

For the NAM mapping population, two replicates of each genotype were included in round 1 and three replicates of each genotype were included in round 2 in a randomized complete block design for each round. Seeds were planted in 4-inch plastic pots and grown 10 days in the Cape Fear Community College horticulture department greenhouse in Castle Hayne, North Carolina. At the three-leaf stage, plants were photographed and measured and stress treatments were begun. Water was withheld from drought plants for 5 d, whereas the remaining plants were watered every other day to full water weight. UV radiation was applied from UV313 bulbs. Pots containing soil but no plants were used to measure water loss rates and final percent water weight. Plants were watered again and allowed to recover for 4 d after stress and then measured. Plant height (to canopy top) was recorded from plant photographs before and after the stress treatment. After treatment, above-ground plant tissue and root biomass were dried and weighed.

#### Data analysis of IBM94 population:

##### Variance components heritability estimation:

Heritability for the IBM94 data set was calculated as previously described for recombinant inbred lines (Holland and Coles 2011; Holland *et al.* 2003). The SAS code for this calculation is provided in Supporting Information, File S1.

##### QTL analysis:

Mixed-model analysis can be scaled to increasingly complex experimental and field designs (van Eeuwijk *et al.* 2010). We chose the mixed-model framework for our data analyses to provide suitable sensitivity and specificity for our comparison of genetic architecture across defined, factorial, controlled stress environments. Our approach is similar to the mixed-model approaches used for density stress in the IBM population (Gonzalo *et al.* 2006) and in a barley example (Malosetti *et al.* 2004), except that we included a combined stress as well as single-stress environments. We considered the allele–environment interaction term as random so that we could estimate the variance for this term to use in our comparison of allele effect sizes. The intermated structure of the IBM population, and the fact that nearly every recombination event that occurred during population development is tagged in the dense marker set, provided us with the ability to cluster correlated markers to increase detection of QTL. This grouping of correlated markers increased the power to detect QTL, at the cost of decreasing the resolution by broadening the map interval. Marker genotypes for linear modeling included the full set of 4678 markers publicly available for the IBM94 RILs (www.maizegdb.org). To allow comparison to other work, the marker map locations from the IBM2 Neighbors 2005 map build (Schaeffer *et al.* 2006) were used for all analyses. Markers with low P values were examined for artificial coinheritance as described previously (Williams *et al.* 2001). Mixed models were fit using SAS 9.2 and SAS Proc PSmooth from SAS Genetics version 2.2 (SAS, Cary, NC). We fit the experimental data using a randomized complete block structure, which incorporated the interaction block effect with the marker–treatment interaction into one error term, because the block effect was not our main focus. The block effect for these data was examined by Richbourg (2008) and did not contribute substantially to the variance explained.

Smoothing across markers adjacent to each other on the chromosome increases our ability to detect QTL, at the cost of broadening the QTL interval (Morrison *et al.* 2010). To perform smoothing, raw P values for each marker from the mixed model were sorted by chromosome location. As suggested by Sun *et al.* (2006), we used the rank method (Zaykin and Zhivotovsky 2005) to transform P values before smoothing, with multiple test correction using the false discovery rate. The SAS code for our analyses is provided in File S7. The P values of type III sum of squares based on F-tests for the marker by environment interaction terms were obtained for each marker. Once the P values obtained from the mixed model were assigned fractional ranks, they were ordered by the location of the marker along the chromosome and adjacent marker P values combined using Proc PSmooth. The Proc Mixed model was then used to reanalyze the markers using the same model structure, with markers that were clustered into a certain location combined as a unified representation. The interaction terms were compared and contrasted using the Estimate and Contrast statement in Proc Mixed. In addition, least-square means, SEs, and 95% CIs were obtained from the mixed model; allelic effect estimates of which allele state confers an increase or decrease in the measured trait are key for comparisons across treatments and for choosing desirable alleles for breeding (Lynch and Walsh 1998), and these effect estimates were an essential feature of our genetic architecture pattern comparison.

### Data analysis of NAM populations

For the NAM data set, model selection for significant marker population stress treatment was performed using modifications of the joint stepwise regression SAS code (Buckler *et al.* 2009) provided by Dr. Peter Bradbury of the USDA-ARS/Cornell University (http://www.maizegenetics.net/images/stories/interests/statgen/model_selection_and_scan.txt). Thresholds for marker inclusion in the model (SLE) are critical in controlling type I and type II errors. We generated simulated data with five QTL of effect sizes ranging from 50% to 8% using the marker set and population structure from our experiment and performed 200 simulations for a range of SLE thresholds. The best balance between type I and type II error rates was found with SLE of 0.001, which had a positive predictive value of 0.9 (Table S1) and a sensitivity of 0.99, corresponding to an FDR of 10%. We thus used SLE of 0.001 for our data analysis. Allele effects were estimated with SAS PROC MIXED, as for the IBM data analysis. SAS and R code for our analyses are provided in File S7.

## Results

### IBM mapping population

#### Change in plant height:

**Figure 2:**
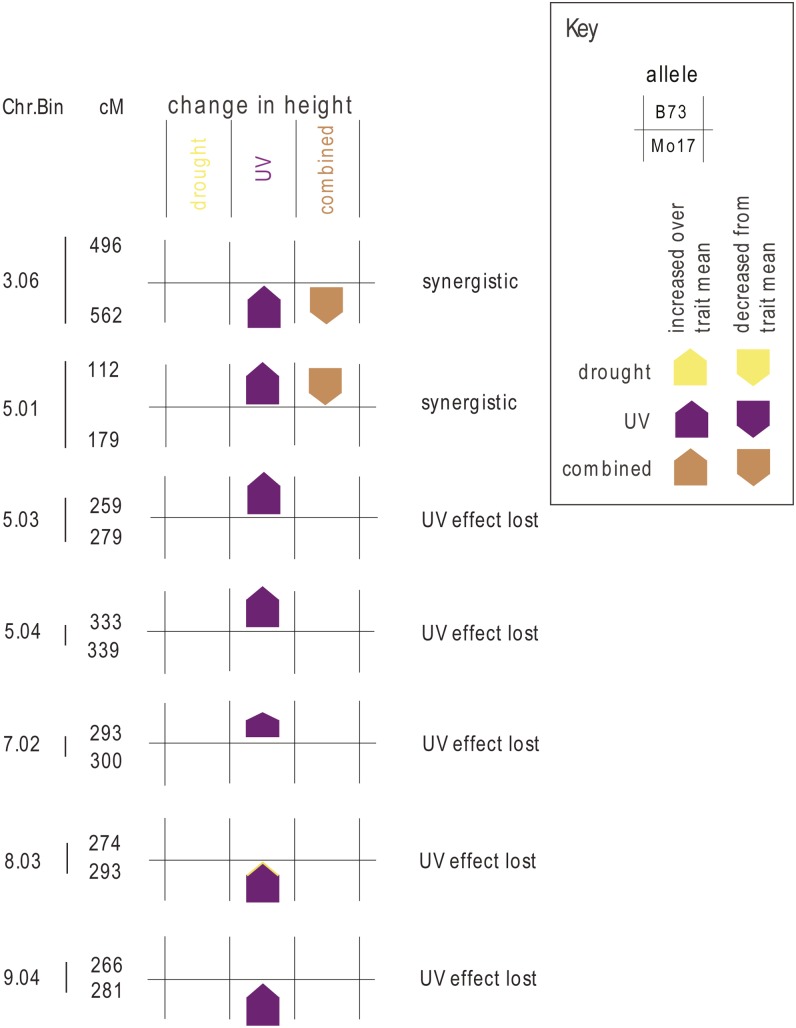
Allele effects of loci important for plant height. Loci with significant genotype by stress treatment interaction for plant height difference (after stress treatment value minus before stress treatment value) are indicated with the chromosome and chromosome bin at left, and then region within the chromosome indicated to the right of the bar in centimorgans (cM). The position of the colored box indicates the responsive allele, with B73 on the top and Mo17 on the bottom for each QTL, and arrows indicate whether the allele effect increased or decreased plant growth as compared to the population mean in each environment. The size of the arrow is proportional to the allele effect size. Only allele effects significantly different than zero are shown.

Seven loci on five different chromosomes were important for explaining environmental interactions for plant height difference (Figure 2). The estimated allele effects for the B73 and Mo17 alleles in each environment that were not significantly different from the population mean are excluded from Figure 2 for ease of viewing; graphs of all the allele effects with their SEs are provided in File S1. Loci important for variation in plant height in the control environment are also shown in File S1. These control no-stress loci do not overlap with the stress-specific QTL shown in the figures.

Two loci important for change in plant height have a UV-responsive allele that is not important in explaining plant height difference in the combined UV and drought treatment. This is indicated in Figure 2 as “synergistic.” The five other UV-responsive QTL, in bins 5.01, 5.03, 7.02, 8.03, and 9.04, have a significant allele effect in the UV environment and no allele effect in the combined stress, and are indicated in Figure 2 as “UV effect lost.” It is apparent from Figure 2 that UV-responsive QTL tend to increase plant growth under that stress and that all the alleles in this group have similar patterns of stress-specific effects whether the allele at that locus is inherited from the B73 or the Mo17 parent.

The change in plant height QTL in bin 5.03 (Figure 2) overlaps with a QTL important for leaf biomass under standard control growth conditions (File S1). The probability of overlap was calculated as in Balint-Kurti *et al.* (2010) and the P value was 0.12, suggesting that this degree of overlap occurs by chance.

#### Leaf biomass:

**Figure 3:**
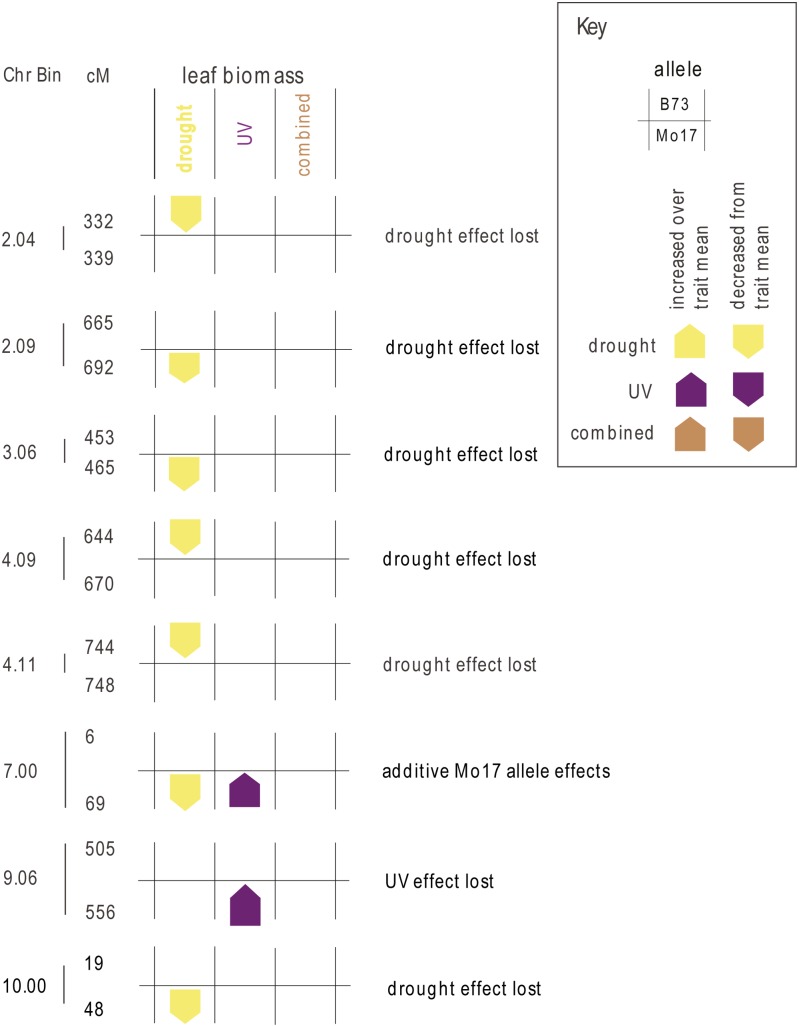
Allele effects of loci important for leaf biomass. Loci with significant genotype by stress treatment interaction for leaf dry weight are indicated with the chromosome and chromosome bin at left, and then region within the chromosome indicated to the right of the bar in centimorgans (cM). The position of the colored box indicates the responsive allele, with B73 on the top and Mo17 on the bottom for each QTL, and arrows indicate whether the allele effect increased or decreased leaf biomass as compared to the population mean in each environment. The size of the arrow is proportional to the allele effect size. Only allele effects significantly different from zero are shown.

Eight loci on six different chromosomes had a significant locus by stress interaction effect for the leaf dry weight trait (Figure 3). The allele effect estimates for each environment at each locus for each allele are provided in File S1. Loci important for variation in leaf biomass in the control environment are also shown in File S1; three control loci, in bins 1.10, 2.07, and 5.05, overlapped with stress-specific QTL. The overlapping loci were removed from further consideration of stress effects and are not shown in Figure 3. The stress-specific QTL in bin 7.00 (Figure 3) showed additive, opposite allele effects, with the Mo17 allele conferring decreased leaf biomass in drought and increased leaf biomass in UV, with no effect under combined stress, as would be predicted from summing the individual stress response effects. The QTL in bin 9.06 exhibited a Mo17 allele UV-responsive allele that was not present in the combined stress treatment. The remaining seven QTL all showed stress-responsive allele effects that were not significantly different from zero in the combined stress environment. Six of these loci had a drought-responsive allele, with four from the B73 parent (shown in upper half of QTL regions in Figure 3) and two with the drought-responsive Mo17 allele (shown in the lower half of the QTL regions in Figure 3). For leaf biomass, the drought-responsive allele tended to decrease plant growth, in contrast to the UV-responsive alleles for leaf biomass (in bin 7.00) and in contrast to the UV-responsive alleles for plant height (Figure 2), which also tended to increase height under UV.

#### Root biomass:

**Figure 4:**
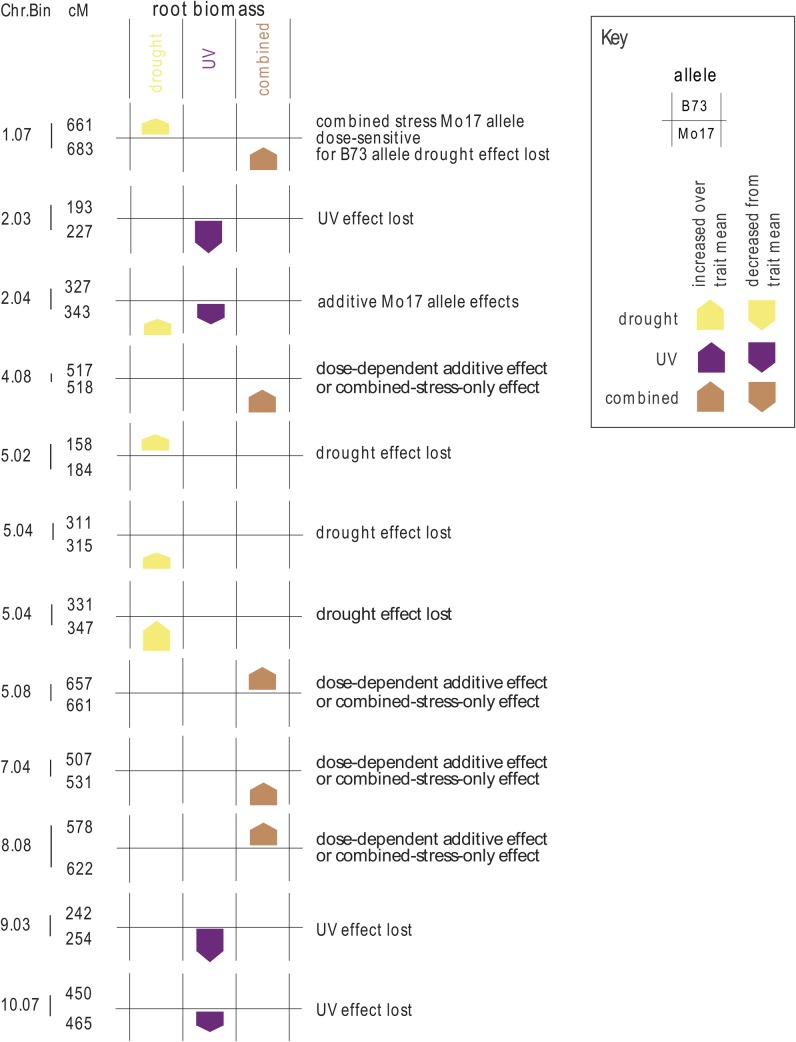
Allele effects of loci important for root biomass. Loci with significant genotype by stress treatment interaction for root dry weight are indicated with the chromosome and chromosome bin at left, and then region within the chromosome indicated to the right of the bar in centimorgans (cM). The position of the colored box indicates the responsive allele, with B73 on the top and Mo17 on the bottom for each QTL, and arrows indicate whether the allele effect increased or decreased plant growth as compared to the population mean in each environment. The size of the arrow is proportional to the allele effect size. Only allele effects significantly different from zero are shown.

Twelve loci on eight different chromosomes had significant locus by stress treatment interactions (Figure 4). Two QTL for root biomass in bin 9.02 and 10.06 colocalized with QTL for root biomass in the control condition, so these were not shown in Figure 4 (see File S1). Bar graphs and SEs for each environment at each locus are shown in File S1, along with loci important for variation in plant height in the control environment. The root biomass trait QTL had five different allelic patterns, with the UV effect lost, drought effect lost, single allele additive, and dose-dependent/additive patterns as seen for plant height and leaf biomass traits. One root biomass locus had a complex allele effect pattern—the QTL in bin 1.07. There was no overall trend for more high or low B73 or Mo17 allele effects in UV or drought for the root biomass trait. Root biomass effects were expected to show a different signaling pattern, because UV radiation was not directly perceived by root tissue. For this trait, additive allele effects loci were more common (Figure 4). This may reflect the length and complexity of the signaling pathway between perception in leaves and effects on root growth.

When the heritability of each trait in each environment was considered individually, there was no consistent pattern of high or low heritability associated with a QTL effect pattern (Table 1). Thus, the pattern of QTL allele effects was not dependent on having relatively high or low amounts of variation explained by the QTL.

**Table 1:** IBM94 variance components for each environment and trait

**Environment**	**Root Biomass**			**Leaf Biomass**			**Change in Height**		
	**Variance (Line)**	**Variance (Error)**	**Line %**	**Variance (Line)**	**Variance (Error)**	**Line %**	**Variance (Line)**	**Variance (Error)**	**Line %**
**Control**	0.12094	0.75083	13.9	0.0024655	0.0088126	21.8	0.04399	25.457	0.17
**Drought**	0.17118	0.62438	21.5	0.0009114	0.01123	7.5	0	18.38979	
**UV**	0.03159	0.82779	3.7	0.0022684	0.01487	13.3	0.89026	19.87988	4.3
**Combined drought and UV**	0.10671	0.79746	11.8	0.0014126	0.01251	11.3	0.73096	19.80387	3.6

### NAM mapping population

#### Plant height difference:

Two QTL were detected in the NAM mapping population for the plant height trait (Table 2). Allele effect estimates for these loci in each environment are provided in File S2 and File S3. For the bin 9.02 Il14H QTL, the sensitive B73 allele conferred decreased growth under UV, with the UV effect no longer detectable in the combined stress treatment. The bin 4.09 QTL was only detected in the combined stress environment and thus was additive or dose-dependent, with the CML277 allele conferring decreased growth when two stress treatments were applied.

**Table 2:** NAM QTL for shoot height difference

**Regulation Pattern**	**Environment**	**Marker**	**Population**	**Significant Allele**	**Allele Effect***^a^* **± SE**	**P Value of Marker**× **Environment**	**Chromosome**	**Location (cM)**	**Bin**
Dose-dependent or combined stress-only effect	Two-stress	PZA01367.2	CML277	CML277	−1.01 ± 0.49	<0.0001	4	112.5	4.09
UV effect lost in combined stress	UV	PZA02344.1	Il14H	B73	−1.07 ± 0.65	0.0016	9	23.8	9.02

a Allele effects are given relative to the population mean, with negative estimates indicating that the allele confers less growth than the average.

#### Root biomass:

Two QTL were detected for root biomass (Table 3). The QTL in bin 4.04/4.05 spanned the centromere, and there was extensive linkage disequilibrium with markers in this region (File S4 and File S5 show marker details). The allele effects were complex, which could be attributable either to multiple coinherited loci in this large region or to one locus with nonadditive allele effects. The bin 10.04 QTL was a dose-dependent/combined stress–only locus, with the M37W allele conferring more root biomass than the B73 allele in the combined stress environment.

**Table 3:** NAM QTL for root biomass

**Regulation Pattern**	**Environment**	**Marker**	**Population**	**Significant Allele**	**Allele Effect***^a^* **± SE**	**P Value of Marker**× **Environment**	**Chromosome**	**Location (cM)**	**Bin**
B73 dose-dependent or combined stress only; CML277 allele single-stress effects lost in combined stress	Two-stress	PZA01422.3 to PZA03231.1*^b^*	CML277	B73	−0.53 ± 0.27	0.0001	4	47.7–57.9	4.04–4.05
	Drought			CML277	0.58 ± 0.27				
	UV			CML277	0.55 ± 0.27				
Dose-dependent or combined stress only effect	Two-stress	PZA01456.2	M37W	M37W	0.55 ± 0.24	0.0016	10	61.6	10.04

a Allele effects are given relative to the population mean, with negative estimates indicating that the allele confers less growth than the average.

b This region includes the centromere of chromosome 4. Markers across the specified region have a consistent pattern of effects and P values. Allele effects and P values are given for the PZA00726.8/10 marker. Detailed information for each marker in this region may be found in the Supporting Information.

As expected, fewer QTL were detected in the NAM populations. There was no evidence for an allelic series (in which alleles from multiple populations have significant effects at a locus and the effects can be sorted by size). The four QTL detected were not in the same locations as in the B73/Mo17 IBM population QTL for those traits, which may be attributable to differences between details of the experimental setup across 2 yr or, more likely, to population-specific allele effects, as for complex traits like growth and yield each population typically has unique genetic effects (Carena *et al.* 2010). However, the pattern of environmental stress factor effects was generally similar to the patterns seen in the IBM population for each trait. For example, the largest effect size plant height QTL (bin 9.04) in the IBM population exhibited “UV effect lost,” as did the largest-effect NAM allele.

## Discussion

For each significant locus in the IBM mapping population (Figure 2, Figure 3, and Figure 4), we examined the prediction from a simple combined signaling theory (Figure 1) and compared the prediction to our observed genetic architecture (Table 4). There were no cases at any locus for the three traits where the independent prediction (Figure 1A) matched the combined stress allele effect (Table 4). In particular, we would expect the largest-effect loci (bin 9.04 in Figure 2, bin 9.06 in Figure 3, and bin 2.03 in Figure 4; details in File S1) to be detectable in both the UV and the combined stress cases if the pattern were independent—but this was not observed. Thus, UV and drought input signaling shared one or more components. This was consistent with the nonadditivity of UV and drought seen in other crops (Mittler 2006).

**Table 4:** Genetic architecture fit to theory

		Signal Interaction	
	Independent Signaling	AND	OR
Theoretical expectations*^a^*	If signals are entirely separate, then there will be no QTL that affects both stresses, so all QTL found in combination stress should also be found in the single-stress condition	The combination QTL will be a mixture of novel loci and loci that are found in single-stress conditions, so at least some single-stress condition QTL should also be seen as a combination stress QTL	The same loci should contain QTL for each stress and for the combination, with the combination allele effect being the sum of the two single allele effects
Observations	This pattern was never seen	A mix of novel and single was observed, but there was no case in which a single-stress QTL was also a combination stress QTL with the same allele effect	Observed in leaf bin 7.00 and in root bin 2.04 IBM QTL

a The expectations from the positive-edge networks displayed in Figure 1 are described in this row.

How are UV and drought stress inputs combined? The OR gate model (Figure 1C) predicted that loci should be significant in all three treatments, although if allele effects were opposite for UV and drought, then an XOR gate would be generated and there would be no significant allele in the combined stress. We observed two XOR patterns (Table 4), one for the leaf biomass trait (Figure 3, bin 7.00) and one for the root biomass trait (Figure 4, bin 2.04). We found no simple OR gate loci; this is consistent with the lack of separate integrator-type plasticity loci in barley mapping experiments with many environments (Lacaze *et al.* 2008). Most of the loci we mapped showed a pattern of allele importance in one stress and no significant effect in the combined stress treatment; these are labeled UV effect lost or drought effect lost in Figure 2, Figure 3, Figure 4, and Table 2. This genetic architecture shared some similarity with the AND gate signaling circuitry (Figure 1B), in that novel loci were detected in combined stress. However, the simple AND gate arrangement also predicted that at least some of the largest-effect alleles should be present and should have a consistent effect direction in the single and combined stress—and we saw no loci with this pattern (Table 4). The AND gate arrangement (Figure 1B) did not explain the synergistic allele effects pattern seen for both plant height and root biomass trait loci. In addition, it was *a priori* unlikely that every possible combination of different abiotic and biotic stress inputs had a separate sensor circuit reserved only for that combination. Consideration of the “effect lost” pattern we observed and the need for parsimony in signaling prompted us to modify our original simple signaling model.

**Figure 5:**
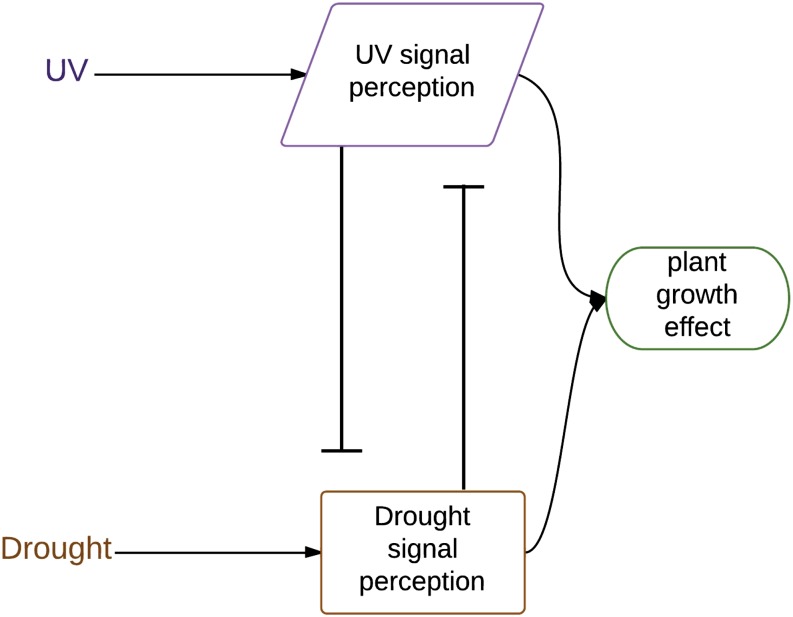
Signaling pathways with constitutive sensors drought and ultraviolet radiation are perceived by sensors that negatively regulate (reduce the effect of) signals from the other environmental input. This mutually negative and attenuating effect is drawn as a direct interaction for simplicity.

Our modified explanation for the prevalence of combined stress–lost loci in our results incorporated a modifier with a blocking effect (Figure 5). Any allelic difference in the UV signaling step would be “invisible” under drought alone, and any allelic effect for the drought signaling step would be undetectable under UV alone. This symbolized the classic modifier locus (Hamilton and Yu 2012) with the additional specification of an attenuating, negative effect on signaling. Signals characterized as blocking, negative, or repressive are often experimentally observed in physiological or regulatory networks. For example, negative edges are prevalent in immune system networks (Campbell *et al.* 2011) and in rice complex trait epistasic networks (Zhang *et al.* 2011). Theoretical analysis of stable network structures also suggested that negative regulation should be favored (Mittenthal and Zou 2011). Recent work regarding metabolic pathways showed that inhibitory pathway interactions are difficult to detect (Blair *et al.* 2012); our approach of manipulating environments provided an alternative detection scheme.

Our analysis of the multiparent NAM population had a more even balance between "combined effect lost" additive one-locus environment effect alleles, as compared to AND gate/negative regulator genetic architectures. If this is seen as a typical pattern in larger better-powered experiments such as those using connected multiparent or diallel mapping populations, then diverse germplasm may be a fruitful source of less complex environmental network interactions. Balanced multiparent populations created from parents with a range of genetic distances, such as sets of AMPRIL populations (Huang *et al.* 2011), would be good candidates for future experimental comparisons of combined stress genetic architectures. Analysis of QTL and of genetic architecture are conflicting goals (Verhoeven *et al.* 2006), and connected multiparent populations can balance these two goals. Theoretical models of the evolution of genetic architecture indicate that the divergence between parents influences the number of potential QTL (Rajon and Plotkin 2013); mathematical modeling incorporating varying and combined environmental inputs as well as optimum subpopulation size and type should be a first step in selecting the best experimental population type and replication structure for follow-up large-scale experiments that would allow statistical comparisons of the patterns of genetic architecture in stress combinations.

It would also be of interest to apply our combined stress experimental design to other abiotic and biotic stress types to determine if other stress combinations are independent or have a modifier-type interaction in generation of the measured phenotype. A qualitative survey of genetic architecture patterns in different stress combinations would be a useful complement to large-scale multiparent population statistical tests for differences in genetic architecture. The pattern of interaction among drought, flooding, nitrogen stress, plant density, and plant disease susceptibility might allow higher-order classification of stress response networks that would support intensive investigation of specific examples and then generalization across similar network types.

Modifiers of allelic effects can be detected through alterations in the expected ratios of progeny classes (Zhang *et al.* 2011), although most quantitative genetics and association experiments have little power to detect such interactions (Carlborg *et al.* 2003; Cordell 2009). It is more common to see modifiers as background effects when defined alleles are "transplanted" into new genomic contexts (Hamilton and Yu 2012); however, it is labor-intensive to identify the allele conferring the newly visible modifier effect in an inbred or ecotype in most model systems. We thus have little information about the types of genes and types of nucleotide changes that act as modifiers. In *Saccharomyces cerevisiae*, systematic analysis of epistasis is possible using deletion collections (Xu *et al.* 2011) and it is possible to perform one-step replacements of alleles identified from QTL mapping experiments (Ehrenreich *et al.* 2012); therefore, examination of combination input (*e.g.*, combination chemical) treatments in yeast may provide a tractable test system for the molecular nature of input-blocking signals and network components and interactions.

The blocking signal network we propose (Figure 5) makes the effects of multiple stresses most predictable in the specific multiple stress environment. This restricted predictive range is seen in crop breeding, in which breeding for desirable traits in high-input environments does not always translate to improvement in low-input settings (Weber *et al.* 2012) despite efforts by breeders to select for environmentally invariant (stable) improved genotypes. Both modeling results (Malcom 2011) and the focus on additive variation in breeding programs (Lynch and Walsh 1998) suggest that small networks of genes are more stable in changing environments. Early screening of diverse populations for small, limited modifier networks may allow more efficient breeding for novel environments. We thus suggest that further modeling of the effects of choosing starting populations from low-input, high-stress environments with the goal of locating populations with small attenuating networks and little variation in modifier alleles would be productive. We recommend modeling of the types of environmental input networks that we described in a breeding simulator such as QuGene (Podlich and Cooper 1998).

The differences between predicted and observed QTL, and the differences between mapping populations, highlight the challenge of locating suitable test environments. Efforts to exploit QTL by environment variability focus on grouping environments using additive–main effect–multiplicative interaction models or genotype–main effect/environment interaction biplots (Gauch *et al.* 2008), and focus on fitting environmental covariates such as temperature to move toward an understanding of the nonlinear/network functions (Messina *et al.* 2011). Any two environments that do have the same QTL, or that have the same underlying parameter QTL for variables such as temperature, thus exhibit the same stress on the plants grown in the environments (van Eeuwijk *et al.* 2010). It may be especially useful to consider locating field experiments in environments of this type, in other words, to use these environments to further query the genetic networks for complex traits. Compilation of QTL experiment raw data and results from commonly used mapping populations along with associated environmental covariates and crop model parameters would be a first step toward choosing informative field sites; unfortunately, field GPS coordinates and planting dates are not typically curated with QTL information but are embedded in individual investigator’s records and are labor-intensive to access. Leveraging of existing mapping and climate information would allow identification of potential independent E signaling inputs more efficiently than testing all possible combinations of managed stresses.

## Supplementary Material

Supporting Information
