# Supplementary material for: Genotype to Phenotype Maps: Multiple Input Abiotic Signals Combine to Produce Growth Effects via Attenuating Signaling Interactions in Maize
Source: G3 (Bethesda). 2013 Oct 18;3(12):2195–204. doi: 10.1534/g3.113.008573 (PMC3852382; doi:10.1534/g3.113.008573)
Supplement: Supporting Information [file supp_3_12_2195__index.html]

Genotype to Phenotype Maps: Multiple Input Abiotic Signals Combine To Produce Growth Effects via Attenuating Signaling Interactions in Maize — Genotype to Phenotype Maps: Multiple Input Abiotic Signals Combine to Produce Growth Effects via Attenuating Signaling Interactions in Maize — Supporting Information 

# Genotype to Phenotype Maps: Multiple Input Abiotic Signals Combine to Produce Growth Effects via Attenuating Signaling Interactions in Maize

## Supporting Information for Makumburage *et al.*, 2013

**Files in this Data Supplement:**

- Supporting Information - Files S1-S7 and Table S1 (PDF, 648 KB)
- File S7 - Supplemental Statistical Methods (PDF, 630 KB)
- Table S1 - Positive predictive value of SLE thresholds for NAM marker selection. (PDF, 348 KB)
- File S1 - IBM raw trait data (.xls, 139 KB)
- File S2 - IBMmarkerset raw data (.csv, 725 KB)
- File S3 - NAM geno (.csv, 10 MB)
- File S4 - NAM map (.csv, 30 KB)
- File S5 - NAM trait data (.csv, 152 KB)
- File S6 - Supplemental Results 1 - IBM Significant Traits (.xlsx, 738 KB)
