# Supplementary material for: Genotype to Phenotype Maps: Multiple Input Abiotic Signals Combine to Produce Growth Effects via Attenuating Signaling Interactions in Maize
Source: G3 (Bethesda). 2013 Oct 18;3(12):2195–204. doi: 10.1534/g3.113.008573 (PMC3852382; doi:10.1534/g3.113.008573)
Supplement: Supporting Information [file supp_g3.113.008573_FileS7.pdf]

File S7
SUPPLEMENTAL STATISTICAL METHODS

Statistical analysis of IBM94 population

Description of data:

This analysis explores the effect of the markers and environments on the growth of corn plants. There are 92 different lines of corn plants. Each line is provided four different environmental conditions (control, drought, UV, drought & UV applied together). The genotype of each recombinant inbred line is determined by 3235 markers. Each marker has its own marker state either A or B, with A symbolizing the B73 parental allele and B symbolizing the Mo17 parental allele. The individual plants within a line shares a common genotypic feature. (i.e. the marker state of a marker is common for all plants within a line which is thus considered as a nested effect). Three types of response variables are recorded from each plant: root biomass, leaf biomass and change in height.

Variance Components for Each Environment Separately

Example SAS code for variance component estimation of heritability for the IBM94 data set (Holland and Coles, 2010; Holland et al 2003) is provided below. This is an estimate of the heritability.

```
proc varcomp data=ibmuvwater method=reml;
      by Env;
      class Line;
      model Trait_3_changeinheight=Line;
      run;
```

QTL Analysis

Mixed models were fit to the IBM94 recombinant inbred line genotype and trait data; these data are provided as supplemental trait and marker files. Marker state (parental allele present for each marker along the chromosome) and environment were fixed effects, with the interaction term (marker state by environment) and the nested effect of markers within lines being random effects. The Proc MIXED in SAS was used for this analysis. Type 111 analysis of variance under Proc MIXED was performed for each marker. The P-value of the interaction term of Type 111 analysis of variance was recorded for each marker. Once all the P-values were obtained from all the markers, a fractional rank of p-values was assigned to each marker. The markers with their fractional ranks were then reordered by the location index of markers along the chromosome. Proc PSMOOTH in SAS was used for grouping of adjacent markers with significant P values as a group, and multiple-test adjustment of the ordered fractional ranks was done using the false discovery rate. After grouping, Proc MIXED was used by group to analyze the group effects with the same random and fixed effects as the initial mixed model fit, with additional Estimate and Contrast statements so that some levels of the interaction term could be compared. Note that the control environment was removed from the whole analysis, and was analyzed separately. The environments are coded as control=1, drought=2, UV=3, and combined drought with UV=4.

SAS Code and Detailed Comments for IBM94

This document first shows the code from the SAS file "ChangeInHeigthForGroupStep2" " in the folder "N:\Stapleton Lab Share\Buddhi\IBM94experiment\FinalOutputIBM94\ Univariate\ChangeInHeight\Env2,3&4". It then gives more comments to the code step by step.

Code from the SAS File

```
libname CHeigth 'N:\Stapleton Lab Share\Buddhi\IBM94experiment\SAS code
\output\2ndIteration\changeinHeight';
```

```
data randomType3;
set CHeigth.randomType3;
if  Source ne 'Env*marker_state'  then delete;
run;
libname IBM94 'N:\Stapleton Lab Share\Buddhi\IBM94experiment\SAS code';

proc sort data=IBM94.markerset out=markerset;
by increment;
run;

data markerset;
   set markerset;
   dummy=_n_;
   run;
   proc sql;
   create table pvalcorlnorder as
   select randomType3.*, markerset.dummy
   from randomType3, markerset
   where markerset.marker= randomType3.marker;
   run;
   quit;
proc rank data=pvalcorlnorder out=pvalcorlnorder2 fraction;
      var probF;
      ranks rankp;
run;
  proc sort data= pvalcorlnorder2;
  by dummy;
  run;
proc psmooth data=pvalcorlnorder2 out=pnew tpm fdr bandwidth=3 to 9 by 2;
      var rankp;
      id dummy;
   run;

   symbol1 v=none i=join;
   legend1 label=none;
    ods listing;

   proc gplot data=pnew;
      plot (rankp rankp_S3 rankp_S5 rankp_S7 rankp_S9)*dummy
           /overlay vref=3.0 legend=legend1;
   run;


data refect1;
set CHeigth.refect;
if Effect ne 'Env*marker_state' then delete;
pot= cats(Env,marker_state);
keep marker Effect Lower Estimate upper pot;
run;
proc transpose data=refect1(keep=marker Effect Lower Estimate upper pot)
out=refect2;
      by marker;
      var Estimate Lower  upper;
run;

data refectEs;
set refect2;
```

```
if _NAME_ ne 'Estimate' then delete;
rename COL1= E2A  COL2= E2B COL3= E3A  COL4= E3B COL5= E4A COL6= E4B ;
run;
data refectLw;
set refect2;
if _NAME_ ne 'Lower' then delete;
rename COL1= L2A  COL2= L2B COL3= L3A  COL4= L3B COL5= L4A COL6= L4B ;
run;
data refectUp;
set refect2;
if _NAME_ ne 'Upper' then delete;
rename COL1= U2A  COL2= U2B COL3= U3A  COL4= U3B COL5= U4A COL6=U4B ;
run;
data refect;
retain marker E2A L2A U2A E2B L2B U2B E3A L3A U3A E3B L3B U3B E4A L4A U4A E4B
L4B U4B;
merge refectEs refectLw refectUp;
by marker;
drop _NAME_;
run;

proc sql;
create table signfcnttmarker as
select pnew.dummy,refect.* , markerset.marker,markerset.increment,
markerset.Chromosome, pvalcorlnorder2.FValue,pvalcorlnorder2.ProbF,
pvalcorlnorder2.rankp
from pnew,pvalcorlnorder2,markerset,refect
where (pnew.dummy = pvalcorlnorder2.dummy)& (pnew.dummy = markerset.dummy)
      & (refect.marker= markerset.marker) ;
run;
quit;
data groupmarker;
set signfcnttmarker;
run;


data groupmarker;
set groupmarker;
if 1133<=dummy<=1160 then group=1;
if 1631<=dummy<=1653 then group=2;
if 1678<=dummy<=1684 then group=3;
if 1702<=dummy<=1716 then group=4;
if 1722<=dummy<=1728 then group=5;
if 1776<=dummy<=1782 then group=6;
if 2059<=dummy<=2065 then group=7;
if 2338<=dummy<=2344 then group=8;
if 2574<=dummy<=2580 then group=9;
if 2583<=dummy<=2589 then group=10;
if 2865<=dummy<=2874 then group=11;
run;
proc export data=groupmarker outfile='N1:\Stapleton Lab
Share\Buddhi\IBM94experiment1
\SAS code\output\2ndIteration\changeinHeight\group\csvFiles\GroupDetails.csv'
replace;
run;

data envi;
```

```
      set IBM94.sheet1;
run;

proc sort data=markerset;
      by marker;
run;
proc transpose data=markerset(keep=marker MO001 - - MO384) out=markertrans;
      by marker;
      var MO001--MO384;
run;

proc sql;
      create table joined as
      select Markertrans.marker,Markertrans._NAME_,Markertrans.COL1 as
marker_state,
      Envi.Trait_3_changeinheight,Envi.Env,Envi.Replicate
      from Markertrans,Envi
      where (Markertrans._NAME_ eq Envi.Line)
      ;
quit;
data joined;
set joined;
if Env eq 1 then delete;
run;

proc sql;
create table subjoined as
select joined.*,groupmarker.group
from joined,groupmarker
where joined.marker=groupmarker.marker;
run;
quit;

proc sort data=subjoined;
by group;
run;


proc mixed data=subjoined  covtest method=type3 ;
      by group;
      where marker_state in ('A','B');
      class Env marker_state  _NAME_ ;

      model Trait_3_changeinheight=    marker_state  /s OUTPRED=predict;

      random _NAME_(marker_state) Env Env*marker_state /s CL ;
      lsmeans marker_state;

      estimate'2A vs 4A' | Env*marker_state  1 0 0 0 -1 0;
      estimate'2B vs 4B' | Env*marker_state 0 1 0 0 0 -1;
      estimate'3A vs 4A' | Env*marker_state 0 0 1 0 -1 0;
      estimate'3B vs 4B' | Env*marker_state  0 0 0 1 0 -1;

      contrast '2A vs 4A' | Env*marker_state  1 0 0 0 -1 0;
      contrast'2B vs 4B' | Env*marker_state 0 1 0 0 0 -1;
      contrast'3A vs 4A' | Env*marker_state 0 0 1 0 -1 0;
      contrast'3B vs 4B' | Env*marker_state  0 0 0 1 0 -1;
```

```
      ods output tests3=tests lsmeans=means SolutionR=REfect SolutionF=FEfect
      CovParms=covtest type3=randomType3 estimates=estmte Contrasts=Contrst;
run;
quit;
libname group 'N1:\Stapleton Lab Share\Buddhi\IBM94experiment\SAS code
\output\2ndIteration\changeinHeight\gr1oup\sasdataset';
data group.tests;
set tests;
run;

data group.means;
set means;
run;
data group.REfect;
set REfect;
run;
data group.FEfect;
set FEfect;
run;
data group.covtest;
set covtest;
run;
data group.Randomtype3;
set Randomtype3;
run;
data group.compareEstmte;
set estmte;
run;
data group.Contarst;
set Contrst;
run;

data forplot;
set refect;
if Effect ne 'Env*marker_state' then delete;
xval= cats(Env,marker_state);
run;

proc export data=forplot outfile='N:\Stapleton Lab
Share\Buddhi\IBM94experiment1
\SAS code\o1utput\2ndIteration\changeinHeight\group\csvFiles\interaction.csv'
replace;
run;



 goptions reset=all  colors=(black blue green red)  ftitle=swissb ;
 symbol1 color=red  value=dot height=.2 ;
ods listing;
ods rtf file = "interactionplot(ChangeInHeight).rtf";
 %macro plot;
%do j=1 %to 11;
 data _null_;
set Groupmarker(where=(group=&j));
by group;
 if first.group then  call symput('first',put(dummy,5.));
```

```
 if last.group then call symput('last',put(dummy,5.));
run;
%if &first=&last %then %let lastname= &last;
%if &first ne &last %then %let lastname= &first to &last;
%put &lastname;

title "Interaction Estimates: group= &j , Location Index: &lastname ";
proc gchart data=forplot(where=(group= &j));
vbar xval  / TYPE=SUM sumvar=Estimate ;
 run;
quit;
 %end;
%mend;
%plot;
ods rtf close;
```

Comments to the code step by step

Code1

```
"libname CHeigth 'N:\Stapleton Lab Share\Buddhi\IBM94experiment\SAS code
\output\2ndIteration\changeinHeight';" -
```

This code assigns the path to the folder "changeinHeight" which contains SAS data sets created by step1.

Code2

```
data randomType3;
set CHeigth.randomType3;
if  Source ne 'Env*marker_state'  then delete;
run;
```

Above code does the following:
Reads in the SAS data set named "randomType3" from the specified folder "changeinHeight" located at 'N:\Stapleton Lab Share\Buddhi\IBM94experiment\SAS code\output\2ndIteration\changeinHeight' and creates another copy of the data "randomType3" with the given condition. The copy is named as "randomType3" and it only has observations where source='Env*marker_state'.

Code3

```
libname IBM94 'N:\Stapleton Lab Share\Buddhi\IBM94experiment\SAS code';
```

This code assigns the path to the folder "SAS code" which contains raw data for analysis.

Code4

```
proc sort data=IBM94.markerset out=markerset;
by increment;
run;
```

Above code does the following:
Reads in the SAS data set named “markerset” from the folder “SAS code” located at “N:\Stapleton Lab Share\Buddhi\IBM94experiment\SAS code” and sorts its data in ascending order by its variable named “increment”. The sorted data is saved as another SAS data set named “markerset” in the SAS work library.
Make sure the SAS data set “markerset” is in the folder “SAS code” with the given path.

Code5

```
data markerset;
   set markerset;
   dummy=_n_;
   run;
```

Above code does the following:
Reads in the SAS data set named “markerset” from the SAS work library and adds another variable named “dummy” to the data and saves it as “markerset” in the SAS work library. The variable “dummy” is used to represent the location index of a marker.

Code6

```
proc sql;
   create table pvalcorlnorder as
   select randomType3.*, markerset.dummy
   from randomType3, markerset
   where markerset.marker= randomType3.marker;
   run;
   quit;
```

Above code does the following:
Reads in all the variables from the data set “randomType3” and the variable “dummy” from the data set “markerset” in the SAS work library and creates another data set named “pvalcorlnorder” from those selected variables whenever the condition “markerset.marker= randomType3” is met. The data set “pvalcorlnorder” has location index and it is used to order fractional ranks for Proc PSMOOTH under Code9.

Code7

```
proc rank data=pvalcorlnorder out=pvalcorlnorder2 fraction;
      var probF;
      ranks rankp;
run;
```

Above code does the following:
Reads in the data set named “pvalcorlnorder” from the SAS work library and creates another SAS data set named “pvalcorlnorder2” in the SAS work library with a new variable named “rankp” which contains the fractional ranks of the variable “probF”.

Code8

```
  proc sort data= pvalcorlnorder2;
  by dummy;
  run;
```

Above code does the following:
Reads in the SAS data set named “pvalcorlnorder2” from the SAS work library and sorts its data in ascending order by its variable named “dummy”. The sorted data is saved again into a SAS data set named “pvalcorlnorder2”.

Code9

```
proc psmooth data=pvalcorlnorder2 out=pnew tpm fdr bandwidth=3 to 9 by 2;
      var rankp;
      id dummy;
   run;
```

Above code does the following:
Reads in the SAS data set named “pvalcorlnorder2” from the SAS work library. Performs multi test adjustments of the fractional rank values using “fdr” algorithm with the given order of the variable named “dummy”. Saves the output from Proc PSMOOTH into a SAS data set named “pnew” in the SAS work library. Note: the PSMOOTH procedure is part of the SAS Genetics package.

Code10

```
   symbol1 v=none i=join;
   legend1 label=none;
    ods listing;

   proc gplot data=pnew;
      plot (rankp rankp_S3 rankp_S5 rankp_S7 rankp_S9)*dummy
           /overlay vref=3.0 legend=legend1;
   run;
```

Above code does the following:
Sets required graphical environment variables to required levels to make plots.
Reads in the SAS data set named “pnew” from the SAS work library. Creates a plot of multi test adjusted fractional rank values.

Code11

```
data refect1;
set CHeigth.refect;
if Effect ne 'Env*marker_state' then delete;
pot= cats(Env,marker_state);
keep marker Effect Lower Estimate upper pot;
run;
```

Above code does the following:
Reads in the SAS data set named “refect” from the library path “CHeigth” assigned by code1. Keeps only the observations whenever the value of the variable “Effect” equals 'Env*marker_state'. Creates another variable named “pot” which represents the levels of the interaction 'Env*marker_state'. Keeps only the variables “marker”, “Effect”, “Lower” ,“Estimate”, “upper” and “pot”; Saves the output from these steps into a SAS data set named “refect1”.

The set of codes (code13 to code 17) is used to create another SAS data set from “refect1” , which is more readable on estimates , lower and upper confidence intervals of random effects.

Code12

```
proc transpose data=refect1(keep=marker Effect Lower Estimate upper pot)
out=refect2;
      by marker;
      var Estimate Lower  upper;
```

```
run;
```

Above code does the following:
Reads in the variables named “marker”, “Effect”, “Lower” ,“Estimate”, “upper” and “pot” from the SAS data set named “refect1” from the SAS work library.
Performs transpose of the variables “Estimate”, “Lower” and “upper” by the variable named “marker”. Saves the output from these steps into a SAS data set named “refect2”.

Code13

```
data refectEs;
set refect2;
if _NAME_ ne 'Estimate' then delete;
rename COL1= E2A  COL2= E2B COL3= E3A  COL4= E3B COL5= E4A COL6= E4B ;
run;
```

Above code does the following:
Reads in the SAS data set named “refect2” from the SAS work library. Keeps only observations whenever the value of the variable “_NAME_” equals to 'Estimate'. Renames the column names. Saves the output from these steps into a SAS data set named “refectEs” in the SAS work library.

Code14

```
data refectLw
set refect2;
if _NAME_ ne 'Lower' then delete;
rename COL1= L2A  COL2= L2B COL3= L3A  COL4= L3B COL5= L4A COL6= L4B ;
run;
```

Above code does the following:
Reads in the SAS data set named “refect2” from the SAS work library. Keeps only observations whenever the value of the variable “_NAME_” equals to 'Lower'. Renames the column names. Saves the output from these steps into a SAS data set named “refectLw” in the SAS work library.

Code15

```
data refectUp;
set refect2;
if _NAME_ ne 'Upper' then delete;
rename COL1= U2A  COL2= U2B COL3= U3A  COL4= U3B COL5= U4A COL6=U4B ;
run;
```

Above code does the following:
Reads in the SAS data set named “refect2” from the SAS work library. Keeps only observations whenever the value of the variable “_NAME_” equals to 'Upper'. Renames the column names. Saves the output from these steps into a SAS data set named “refectUp” in the SAS work library.

Code16

```
data refect;
retain marker E2A L2A U2A E2B L2B U2B E3A L3A U3A E3B L3B U3B E4A L4A U4A E4B
L4B U4B;
merge refectEs refectLw refectUp;
by marker;
```

```
drop _NAME_;
run;
```

Above code does the following:
Reads in the SAS data sets named “refectEs”, “refectLw” and “refectUp” from the SAS work library. Merges data sets by the Variable “marker”. Drops the variable “_NAME_”. Saves the output from these steps into a SAS data set named “refect” in the SAS work library.

Code17

```
proc sql;
create table signfcnttmarker as
select pnew.dummy,refect.* , markerset.marker,markerset.increment,
markerset.Chromosome, pvalcorlnorder2.FValue,pvalcorlnorder2.ProbF,
pvalcorlnorder2.rankp
from pnew,pvalcorlnorder2,markerset,refect
where (pnew.dummy = pvalcorlnorder2.dummy)& (pnew.dummy = markerset.dummy)
      & (refect.marker= markerset.marker) ;
run;
quit;
```

Above code does the following:
Reads in the variables which are listed in the select clause above from the data sets named “pnew”,”pvalcorlnorder2”,”markerset” and ”refect” from the SAS work library. Subsets the selected data with the given conditions from the where clause above. Saves the output from these steps into a SAS data set named “signfcnttmarker” in the SAS work library. The Code17 is used to combine some other important details of the markers found in the data set “pnew”.

Code18

```
data groupmarker;
set signfcnttmarker;
run;
```

Above code does the following:
Makes a copy of the data set “signfcnttmarker” and names it as “groupmarker”. The data set “groupmarker” will be used for grouping markers.

Code19

```
data groupmarker;
set groupmarker;
if 1133<=dummy<=1160 then group=1;
if 1631<=dummy<=1653 then group=2;
if 1678<=dummy<=1684 then group=3;
if 1702<=dummy<=1716 then group=4;
if 1722<=dummy<=1728 then group=5;
if 1776<=dummy<=1782 then group=6;
if 2059<=dummy<=2065 then group=7;
if 2338<=dummy<=2344 then group=8;
if 2574<=dummy<=2580 then group=9;
if 2583<=dummy<=2589 then group=10;
```

```
if 2865<=dummy<=2874 then group=11;
run;
```

Above code does the following:
Reads in the SAS data set named “groupmarker” from the SAS work library.
Creates another variable named “group”. Saves the output from these steps into a SAS data set named “groupmarker” in the SAS work library. When assigning values for the variable “group” the estimates and lower and upper confidence intervals are considered from other variables of the data set “groupmarker”.

Code20

```
proc export data=groupmarker outfile='N1:\Stapleton Lab
Share\Buddhi\IBM94experiment1
\SAS code\output\2ndIteration\changeinHeight\group\csvFiles\GroupDetails.csv'
replace;
run;
```

Above code does the following:
Reads in the SAS data set named “groupmarker” from the SAS work library. Saves a csv file of the data set and names it as “GroupDetails.csv” in the csv foder located at “'N1:\Stapleton Lab Share\Buddhi\IBM94experiment1\SAS code\output\2ndIteration\changeinHeight\group\csvFiles”.

Code21

```
data envi;
       set IBM94.sheet1;
run;
```

Above code does the following:
Reads in the SAS data set named “sheet1” from the specified path under code3[The “SAS code” folder located at “N:\Stapleton Lab Share\Buddhi\IBM94experiment\SAS code”] and creates another copy of the data “sheet1”. The copy is named as “envi” and saved in the SAS work library.

Code22

```
proc sort data=markerset;
       by marker;
run;
```

Above code does the following:
Reads in the SAS data set named “markerset” created by code4 from the SAS work library and sorts its data in ascending order by marker. The sorted data is saved in the SAS work library as “markerset”.

Code23

```
proc transpose data=markerset(keep=marker MO001 - - MO384) out=markertrans;

       by marker;
       var MO001--MO384;
run;
```

Above code does the following:
Reads in the SAS data set named “markerset” from the SAS work library. Only the variables named “marker”,”MO001” to “MO384” will be used due to option “(keep=marker MO001 - - MO384)”. Performs transpose of the column data named”MO001” to “MO384” in to a single column. Output from this transpose is saved to another SAS data set named “markertrans”. The new data set “markertrans” is saved in the SAS work library. The data “markerset” needs to be sorted by marker prior to this transpose; code23 is used to sort data by marker.

Code24

```
proc sql;
      create table joined as
      select Markertrans.marker,Markertrans._NAME_ ,Markertrans.COL1 as
      marker_state,
      Envi.Trait_3_changeinheight,Envi.Env,Envi.Replicate
      from Markertrans,Envi
      where (Markertrans._NAME_ eq Envi.Line)
      ;
quit;
```

Above code does the following:
Reads in the variables Markertrans.marker, Markertrans._NAME_, Markertrans.COL1 , Envi.Trait_3_changeinheight,Envi.Env and Envi.Replicate from the SAS data sets Markertrans and Envi in SAS work library and subsets data with the condition Markertrans._NAME_=Envi.Line. All the observarions which are met the condition will be saved to another SAS data set named “joined” in SAS work library. This code is used to create a data set which represents dependent and independent variables.

Code25

```
data joined;
set joined;
if Env eq 1 then delete;
run;
```

Above code does the following:
Reads in the SAS data set named “joined” from the SAS work library and deletes rows whenever the column “Env” has a value of 1. The data from this step will be saved to the same named “joined” in the SAS work library. Therefore SAS data set named “joined” prior to code26 will be replaced by the data coming from code 26. The Code 26 is used to remove data from environment1.

Code26

```
proc sql;
create table subjoined as
select joined.*,groupmarker.group
from joined,groupmarker
where joined.marker=groupmarker.marker;
run;
quit;
```

Above code does the following:
Reads in all the variables from the data set “joined” and the variable “group” from the data set “groupmarker” from the SAS work library and subsets the observations with the condition “joined.marker=groupmarker.marker”.

The data results from these steps will be saved as a SAS data set named "subjoined" in the SAS work library. The code 27 is used to create dependent and independent variables only for the significant markers listed in the data set "groupmarker".

Code27

```
proc sort data=subjoined;
by group;
run;
```

Above code does the following:
Reads in the SAS data set named "subjoined" from the SAS work library and sorts its data in ascending order by its variable named "group". The sorted data is saved again into a SAS data set named "subjoined". Therefore SAS data set named "joined" prior to code28 will be replaced by the data coming from code 28. Since Proc mixed model in code28 is run by group the input data set for code28 is necessary to sort by group, hence the use of code27.

Code28

```
proc mixed data=subjoined  covtest method=type3 ;
      by group;
      where marker_state in ('A','B');
      class Env marker_state  _NAME_ ;

      model Trait_3_changeinheight=    marker_state  /s OUTPRED=predict;

      random _NAME_(marker_state) Env Env*marker_state /s CL ;
      lsmeans marker_state;


      estimate'2A vs 4A' | Env*marker_state  1 0 0 0 -1 0;
      estimate'2B vs 4B' | Env*marker_state 0 1 0 0 0 -1;
      estimate'3A vs 4A' | Env*marker_state 0 0 1 0 -1 0;
      estimate'3B vs 4B' | Env*marker_state  0 0 0 1 0 -1;

      contrast '2A vs 4A' | Env*marker_state  1 0 0 0 -1 0;
      contrast'2B vs 4B' | Env*marker_state 0 1 0 0 0 -1;
      contrast'3A vs 4A' | Env*marker_state 0 0 1 0 -1 0;
      contrast'3B vs 4B' | Env*marker_state  0 0 0 1 0 -1;

      ods output tests3=tests lsmeans=means SolutionR=REfect SolutionF=FEfect
      CovParms=covtest type3=randomType3 estimates=estmte Contrasts=Contrst;
run;
quit;
```

Above code does the following:
"method=type3" – performs type3 analysis of variance throughout the analysis.

"by group;" – Fits mixed model to each group.

"where marker_state in ('A','B')" - use only observations which has its level either A or B. Observations with missing values for marker state will not be used in the analysis.

“class Env marker_state _NAME_” - The variables “Env”, “marker_state” and “_NAME_” in the input data set is regarded as class variables.

“model Trait_3_changeinheight= marker_state /s ;” – Specifies “Trait_3_changeinheight” to be the dependent variable. Specifies “marker_state” to be Fixed effect variable.

“random _NAME_(marker_state) Env Env*marker_state /s CL” – Specifies the nested effect “_NAME_(marker_state)” to be random effect. Specifies “Env” to be random effect. Specifies “Env*marker_state” to be random effect. Requests confidence intervals to be calculated for random estimates.

“estimate'2A vs 4A' | Env*marker_state 1 0 0 0 -1 0;
estimate'2B vs 4B' | Env*marker_state 0 1 0 0 0 -1;
estimate'3A vs 4A' | Env*marker_state 0 0 1 0 -1 0;
estimate'3B vs 4B' | Env*marker_state 0 0 0 1 0 -1;” - makes comparisons between levels of the interaction. For eg; the first estimate statement compares observations coming from environment2 and marker state A with the observations coming from environment4 and marker state A.

“contrast '2A vs 4A' | Env*marker_state 1 0 0 0 -1 0;
contrast'2B vs 4B' | Env*marker_state 0 1 0 0 0 -1;
contrast'3A vs 4A' | Env*marker_state 0 0 1 0 -1 0;
contrast'3B vs 4B' | Env*marker_state 0 0 0 1 0 -1;” - makes contrasts between levels of the interaction. For eg; the second contrast statement makes contrast of the observations coming from environment2 and marker state B versus the observations coming from environment4 and marker state B.

“ods output tests3=tests lsmeans=means SolutionR=REfect SolutionF=FEfect CovParms=covtest type3=randomType3 estimates=estmte Contrasts=Contrst;” – Requested SAS data sets(“tests”, “means”, “REfect” ,”FEfect” ,”covtest”, “randomType3”, “estmte” and “Contrst”) will be created in the SAS work library from the mixed model analysis. The data set named “randomType3” has the details from type 3 analysis of variance. The data set named “REfect” has the details of random effect estimations and their confidence intervals. The data set named “estmte” has the details of comparisons between levels of the interaction term. The data set named “Contrst” has the details of contrasts between levels of the interaction term.

Code29

“libname group 'N1:\Stapleton Lab Share\Buddhi\IBM94experiment\SAS code \output\2ndIteration\changeinHeight\gr1oup\sasdataset';” – Assigns a path to save SAS data sets.

Code30

```
data group.tests;
set tests;
run;
```

Above code does the following:
Reads in the SAS data set named “tests” from the SAS work library and saves it on the location specified by code 29 as another SAS data set named “tests”.

Code31

```
data group.means;
set means;
run;
data group.REfect;
set REfect;
run;
data group.FEfect;
set FEfect;
run;
data group.covtest;
set covtest;
run;
data group.Randomtype3;
set Randomtype3;
run;
data group.compareEstmte;
set estmte;
run;
data group.Contarst;
 set Contrst;
run;
```

Above code does the following:
Saves SAS data sets on the location specified by code29.

Code32

```
data forplot;/*detects only interaction estimates for plots*/
set refect;
if Effect ne 'Env*marker_state' then delete;
xval= cats(Env,marker_state);/*identifies the level of interaction*/
run;
```

Above code does the following:
Reads in the SAS data set named "refect" from the SAS work library and keeps only the observations whenever the variable "Effect" equals to "'Env*marker_state". Creates another variable named "xval" which represents the levels of the interaction. The data results from these steps will be saved as a SAS data set named "forplot" in the SAS work library.

Code34

```
proc export data=forplot outfile='N:\Stapleton Lab
Share\Buddhi\IBM94experiment1\SAS code\o1utput\2ndIteration
\changeinHeight\group\csvFiles\interaction.csv' replace;
run;
```

Above code does the following:
Reads in the SAS data set named "forplot" from the SAS work library and creates a csv file of it in the folder "csvFiles" located at "'N:\Stapleton Lab Share\Buddhi\IBM94experiment1
\SAS code\o1utput\2ndIteration\changeinHeight\group\csvFiles".

Code35

```
goptions reset=all  colors=(black blue green red)  ftitle=swissb ;
 symbol1 color=red  value=dot height=.2 ;
ods listing;
ods rtf file = "interactionplot(ChangeInHeight).rtf";/*creates word document
of plots*/
```

Above code does the following:

Sets graphic environments to required levels to create plots. Requests the output of plots to be written to a rtf file.

Code36

```
 %macro plot;/*generates plots */
%do j=1 %to 11;/*creates plots for all groups at once through the loop*/
data _null_;
set Groupmarker(where=(group=&j));
by group;
 if first.group then  call symput('first',put(dummy,5.));
 if last.group then call symput('last',put(dummy,5.));
run;
%if &first=&last %then %let lastname= &last;
%if &first ne &last %then %let lastname= &first to &last;
%put &lastname;

title "Interaction Estimates: group= &j , Location Index: &lastname ";
proc gchart data=forplot(where=(group= &j));
vbar xval  / TYPE=SUM sumvar=Estimate ;
 run;
quit;
 %end;
%mend;
%plot;
ods rtf close;/*finishes word document of plots*/
```

Above code does the following:

The do loop inside the macro code allows to read in observations only from a group at a time out of 11 groups. The data read is then directs to proc gchart and creates a plot for the group. As the do loop iterates, 11 plots will be created. The last statement "ods rtf close;" opens the created rtf file of 11 plots.

Estimated allele effects for significant loci identified with this method were plotted with standard error bars (Supplemental Results 1.xlsx). Any locus with only a single marker significant was removed from further consideration. These loci are marked with crosshatches and text notes in the Supplemental Results 1 file.

<u>Statistical analysis of NAM population</u>

Part 1 Data Analysis

<u>Overview and Explanation of Mixed model for NAM study:</u>

Description of data

A subset of 50 RIL lines from five different parent populations was used. The first round of the experiment was done in June 2009, with two replicates of each RIL*treatment, and the second round of the experiment was done in July 2009, with three replicates of each RIL*treatment, where the treatment has five different levels. Root and leaf biomass and change in plant height were measured. The genotypic feature of a plant is determined by a set of 1106 markers.

Statistical analysis overview

The analysis for June and July samples was performed together. As the first step of this analysis missing data was imputed using EM algorithm of Proc MI in SAS. Each marker from the set of markers (1106 markers) was considered as covariate rather than as classification variable. In order to determine important markers from the set of markers a model selection was performed using Proc GLMSELECT in SAS. The class variables, treatment and population, are forced to stay in the model as independent variables throughout the model selection. The three way interaction terms, treatment by population by markers, were also present as independent variables during the model selection. The dependent variable was set to be one of the three response variables, each of which is analyzed separately. The significance level for entry into a selection model was predetermined to be 0.001 and significance level for staying in a selection model was also set to be 0.0002 as described by P. Bradbury..

Once the GLM selection is done, a mixed model is then fit for each marker (referred as scan marker) with a background selection process from the markers which are included in the final GLM selection (The set of final GLM selected markers is referred as qualified set). This selection process is based on the chromosome and position of the scan marker. The following rules determine elimination criteria of the markers from the qualified set with regard to a scan marker.

(1) A marker from the qualified set is excluded if it has the same chromosome as for the scan marker.
(2) If relative distant of the position between a marker in the qualified set and the scan marker is less than or equal to 20 map units, then the marker from the qualified set is excluded.

Once scan marker process finished the selection process from the qualified set, a Proc MIXED model was fit for the final marker set (each scan marker with its selected markers). The variables, treatment and population, were considered as the fixed effects. Three way interaction terms, treatment by population by markers in the final marker set, were considered as the random effects. The dependent variable chosen in the GLMSELECT model is the dependent variable for the mixed model. Type 3 analysis of variance was performed for the mixed model. The P-value of the three way interaction term of the scan marker from Type 111 analysis of variance was recorded. This process continued until all the markers become scan markers.

Overview of SAS code used to run Proc GLMSELECT

Part 1 Marker selection and effect estimation for NAM population analyses

The data set 'alldatasub' contains all the dependent and independent variables.
The class variable 'treatment' has four levels: B, C, D and U (B=combined UV and drought stress, C=control, D=drought, and U=ultraviolet radiation)
The class variable 'pop' has five levels: 5,10,11,16 and 22.
The dependent variable, 'plantheight', represents change in plant height.
The macro variable 'factor' is resolved to "treatment pop treatment*pop*m1 to treatment*pop*m1106", where m1 represents marker1 and m1106 represents marker1106.
The option, 'include=2', forces the first two independent variables in the model statement to stay in the model throughout the selection process.

The following SAS code is used in Proc MIXED with the Proc GLMSELECT statements for the dependent variable change in plant height.

```
proc mixed data=alldatasub method=type3 ;
class treatment pop;
model plantheight = treatment pop;
random &modelRandom /s;
ods output "Fit Statistics"=full SolutionR=RdmEffect type3=randomType3;
```

```
run;
```

The data set ‘alldatasub’ contains all the dependent and independent variables.
The class variable ‘treatment’ has four levels: B, C, D and U (B=combined UV and drought stress, C=control, D=drought, and U=ultraviolet radiation)
The class variable ‘pop’ has five levels: 5,10,11,16 and 22.
The dependent variable, ‘plantheight’, represents change in plant height.
The option, ‘method=type3’ , performs type 3 analysis of variance.
The value of the macro variable, ‘modelRandom’, is dependent on the scan marker. For the scan marker1 the macro variable, ‘modelRandom’, is resolved to ‘treatment*pop*m56 treatment*pop*m389 treatment*pop*m391 treatment*pop*m1’.

SAS Code and Detailed Comments for NAM Analysis

SAS code for change in height

```
options nonotes nosource nosource2 errors=0;

proc printto log="N:\Stapleton Lab Share\Buddhi\NAM
info\NAMjj\FinalOutputjjNAM\ChangeInHeight\jjsasdatafromcode\filename.log";
run;
proc printto print="N:\Stapleton Lab Share\Buddhi\NAM
info\FinalOutputjjNAM\ChangeInHeight\jjsasdatafromcode\filename.1st";
run;



/*specifies path to the reponse data set*/
libname rawdata 'N:\Stapleton Lab Share\Buddhi\NAMexperiment\NAM raw data';

data response;/*imports response variables from jj sample*/
set rawdata.sheetnamjj2;
run;
/*specifies path to the genotype and mapping data sets*/
libname Map 'N:\Stapleton Lab Share\Buddhi\NAMexperiment\NAM genotypes';
data markerDetails;/*imports genotype data set*/
set Map.geno;
run;

proc sql;/*combines response variables with marker details */
create table alldata as
select response.*,markerDetails.*
from response,markerDetails
where markerDetails.line=response.line;
quit;

data alldatasub;/*creates a new response variable for change in plant
height*/
set alldata;
plantheight= after_plant_height__cm_ - before_plant_height__cm_;
run;
proc means data=alldatasub N nmiss;/*checks for missing data in response
variables*/
var root_biomass__g_ shoot_biomass__g_ plantheight;
run;
```

```
proc means data=alldatasub N nmiss;/*checks for missing data in set of
markers*/
var m1-- m1106;
run;

   proc mi data=alldatasub seed=21355417 nimpute=1 OUT=alldatasub;/*imputes
missing
                                                          data using EM
algorithm*/
      EM;
      var root_biomass__g_ shoot_biomass__g_ plantheight m1 ;
   run;
proc means data=alldatasub N nmiss; /*checks further if missing data is
present*/
var root_biomass__g_ shoot_biomass__g_ plantheight;
run;
%let factor = treatment pop ;/*introduces a macro variable to use in
GLMSELECT */
%macro makefactor; /*assings required text to the macro varible through a
loop*/
      %do i = 1 %to 1106;
            %let factor = &factor treatment*pop*m&i;
      %end;
%mend;
%makefactor;/*executes macro code of makefactor*/
%put &factor; /*writes assigned text of the macro variable factor to the
log*/

proc glmselect data=alldatasub;/*runs model selections to determine important
markers*/
class treatment pop ;
model plantheight =  &factor /INCLUDE=2 select=sl sle=0.001 sls=2e-4 stop=50;
run;

data modelterms;/*creates a data set from important markers detected in
GLMSELECT*/
length name $5;
input mnum;
name = 'm'||left(mnum);
chr = 0;
pos = 0;
cards;

23
525
526
;
run;

data map;/*imports map data set into SAS from the given library path Map*/
set Map.map;
run;

proc sql;/*assigns chromosome and position to the makers in the data set
modelterms*/
update modelterms set chr=(select chr from map where
modelterms.name=map.name);
```

```
update modelterms set pos=(select pos from map where
modelterms.name=map.name);
quit;


%global modelRandom;/*introduces another global macro variable*/
%global FxdMarker;
%macro markerselection(marker, window);/*performs the marker selection for
the scan marker
                                                        from the markers in
the modelterms*/
      %let mname = m&marker;
      proc sql;/*detects chromosome and position of the scan marker*/
      select chr,pos into :chr,:pos from map where map.name="&mname";
      quit;

      data _null_1;
      set modelterms end=stop;
      length modelRandom $1000;
      length FxdMarker $1000;

      retain modelRandom ;
            retain FxdMarker ;
      if chr^=&chr then /*specifies a requirement for the selection process*/
            do;
          /*adds required text to the data set variable model random when the
condition is met*/
              modelRandom = trim(modelRandom)||" treatment*pop*"||left(name);
              FxdMarker = trim(FxdMarker)||"" ||left(name);
            end;
      else if abs(pos - &pos) > &window then/*specifies a requirement for the
selection process*/
            do;
             /*adds required text to the data set variable model random when
the condition is met*/
                  modelRandom = trim(modelRandom)||"
treatment*pop*"||left(name);
                  FxdMarker = trim(FxdMarker)||""||left(name);
            end;
      if stop then /*checks if last observation is reached*/
            do;
                  call symput('modelRandom',trim(modelRandom));/*assings the
last value of the data
                                                                set variable
modelRandom
                                                                 to the macro
variable modelRandom*/

                  call symput('FxdMarker',trim(FxdMarker));
            end;
      run;
%mend;

%macro fitmodel(marker, window);/*performs a mixed model to the scan marker*/

      %markerselection(&marker, &window);/* Executes the macro code of
markerselection
```

```
                                          for the scan marker */

      %let modelRandom = &modelRandom treatment*pop*m&marker;/*updates text
of modelRandom with
                                                         reauired text from
the scan marker*/

      %let FxdMarker= &FxdMarker m&marker;
      %put calculating the full model for m&marker;

      proc mixed data=alldatasub method=type3 ;/*executes proc mixed */
      class treatment pop m&marker;
      model plantheight = treatment pop &FxdMarker /s ;
      random &modelRandom /s;
      ods output "Fit Statistics"=full SolutionR=RdmEffect type3=randomType3
SolutionF=FEfect;
      run;


      data RdmEffect;/*modifies RdmEffect data set to get required details*/
      set RdmEffect;
       rename m&marker=MarkerLevel;
      if Effect eq "treatment*pop*m&marker." ;
      marker= input("&marker", 5.);
      run;

      data randomType3;/*modifies randomType3 data set to get required
details*/
      set randomType3;
      marker= input("&marker", 5.);
      run;

      data FEfect;/*modifies fixeffect data set to get required details*/
      set FEfect;
      rename m&marker=MarkerLevel;
      marker= input("&marker", 5.);
      run;



     %if &marker eq 1 %then %do; /*adds required details to the Pval data set
from
                                     RdmEffect data set as it scans different
markers */
              data pval;
              set RdmEffect;
              run;
          %end;
         %else %do;
          data pval;
              set pval RdmEffect;
              run;
            %end;


   %if &marker eq 1 %then %do;/*adds required details to the randomType3T1
data set from
```

```
                                        randomType3T data set as it scans
different markers */
              data randomType3T1;
              set randomType3;
              run;
          %end;
         %else %do;
          data randomType3T1;
              set randomType3T1 randomType3 ;
              run;
            %end;



     %if &marker eq 1 %then %do; /*adds required details to the Pval data set
from
                                        RdmEffect data set as it scans different
markers */
              data FEfectAll;
              set FEfect;
              run;
          %end;
         %else %do;
          data FEfectAll;
              set FEfectAll FEfect;
              run;
            %end;


%mend;

%macro scanmarkers(start, finish, window);/*declares starting, ending and
relative distance
                                                    to perform the scan */

      %do i = &start %to &finish;/*scans all the markers one at a time */
            %fitmodel(&i, &window);/* Executes the macro code of fitmodel for
the scan marker*/
      %end;
%mend;

%scanmarkers(1,1106,20);/*sets values for starting, ending and relative
distance and runs
                          the macro code of scanmarkers */


/*Eventhough the last three major macro codes are in the order of
Markerselection,
 fitmodel and scanmarkers, they are executed from the oposite order of
scanmarkers,
fitmodel and Markerselection due to the nested structure of coding*/


proc sort data=pval;/*sorts data set pval by marker*/
by marker;
run;
proc sort data=Randomtype3t1;/*sorts data set Randomtype3t1 by marker*/
```

```
by marker;
run;

proc sort data=FEfectAll;/*sorts data set Randomtype3t1 by marker*/
by marker;
run;

libname results 'N:\Stapleton Lab Share\Buddhi\NAM
info\NAMjj\FinalOutputjjNAM\ChangeInHeight\jjsasdatafromcode';/*specifies
path to save important sas
                                         data sets created by proc mixed and
proc sql*/

data results.Estimatespval;/* saves pval data set on the location*/
set pval;
run;
data results.Randomtype3Pval;/* saves Randomtype3t1 data set on the
location*/
set Randomtype3t1;
run;

data results.FEfectAll;/* saves Randomtype3t1 data set on the location*/
set FEfectAll;
run;



proc printto;
run;

options notes source source2 errors=20;
```

Note: leaf biomass and root biomass traits were analyzed using appropriate modifications to this code for each of the traits.

Part 2 Simulations to determine best NAM model selection SLE threshold

R code for simulations

```
## Read data set in ##

datC<-read.table("H:/Stapleton-Project/GenoAna.trait_Geno_control.txt",sep="",header=TRUE)

datD<-read.table("H:/Stapleton-
Project/GenoAna.trait_Geno_Drought.txt",sep="",header=TRUE)

datU<-read.table("H:/Stapleton-Project/GenoAna.trait_Geno_UV.txt",sep="",header=TRUE)
```

```
datB<-read.table("H:/Stapleton-Project/GenoAna.trait_Geno_Both.txt",sep="",header=TRUE)

## Get rid of possible data frame ##
pure.datC<-(do.call(cbind,datC)); colnames(pure.datC)<-NULL; CstemZ<-pure.datC[,8];
pure.datD<-(do.call(cbind,datD)); colnames(pure.datD)<-NULL; DstemZ<-pure.datD[,8];
pure.datU<-(do.call(cbind,datU)); colnames(pure.datU)<-NULL; UstemZ<-pure.datU[,8];
pure.datB<-(do.call(cbind,datB)); colnames(pure.datB)<-NULL; BstemZ<-pure.datB[,8];

## Obtaining Stem data set for each treat ##
CstemZ=pure.datC[,8]; DstemZ=pure.datD[,8];UstemZ=pure.datU[,8];BstemZ=pure.datB[,8];
nC=length(CstemZ);nD=length(DstemZ);nU=length(UstemZ);nB=length(BstemZ);

## Data ploting ##
op <- par(mfrow = c(2, 2), # 2 x 2 pictures on one plot;
        pty = "s")      # square plotting region; independent of device size;
plot((1:nC),sort(CstemZ));
plot((1:nD),sort(DstemZ));
plot((1:nU),sort(UstemZ));
plot((1:nB),sort(BstemZ));
## At end of plotting, reset to previous settings:
par(op); dev.off();


## QQNorm for Stem for each treat ##
op <- par(mfrow = c(2, 2), # 2 x 2 pictures on one plot;
```

```
    pty = "s")      # square plotting region; independent of device size;
qqnorm(CstemZ);
qqnorm(DstemZ);
qqnorm(UstemZ);
qqnorm(BstemZ);
par(op); dev.off();


## Now we want to input the genotype data ##
## 1-treat; 2-line; 3-replica; 4-rootZ; 5-shootZ; 6-leafZ; 7-heightZ; 8-stemZ; 9-pop; 10-1115: QTL1-QTL1106.
C_Xmatrix=pure.datC[,10:1115];
D_Xmatrix=pure.datD[,10:1115];
U_Xmatrix=pure.datU[,10:1115];
B_Xmatrix=pure.datB[,10:1115];

Cpop=pure.datC[,9];
Dpop=pure.datD[,9];
Upop=pure.datU[,9];
Bpop=pure.datB[,9];

## I try to look at Drought data first ##
X=D_Xmatrix;n=nD

m=1106 ## numberof markers
```

```
## I look at drought first ##


##~~~~~~~~~First 50 Replica ~~~~~~~~~~~~~~~~##
randsample=NULL; randsampleORIG=NULL; AA=NULL;

set.seed(200);
for (i in 1:10)
{
BB=NULL;
e=rnorm(nD)
sm=sample(1:m, 3, replace = FALSE)
i=sm[1]; j=sm[2]; k=sm[3]; print(c(i,j,k));
randsampleORIG=rbind(randsampleORIG, c(i, j, k));
randsample=rbind(randsample, sort(sm))
y=35+3*X[,sm[1]] +5*X[,sm[2]]+ X[,sm[3]] +e
BB=cbind(y,Dpop, X, rep(sm[1], n), rep(sm[2], n),  rep(sm[3], n))
AA=rbind(AA,BB);
}
print(randsample);
seed200=randsample;print(seed200);

set.seed(100);
for (i in 1:10)
{
```

```
BB=NULL;
e=rnorm(nD)
sm=sample(1:m, 3, replace = FALSE)
i=sm[1]; j=sm[2]; k=sm[3]; print(c(i,j,k));
randsampleORIG=rbind(randsampleORIG, c(i, j, k));
randsample=rbind(randsample, sort(sm))
y=35+3*X[,sm[1]] +5*X[,sm[2]]+ X[,sm[3]] +e
BB=cbind(y,Dpop, X, rep(sm[1], n), rep(sm[2], n),  rep(sm[3], n))
AA=rbind(AA,BB);
}
print(randsample);
seed100=randsample;print(seed100);

set.seed(110);
for (i in 1:10)
{
BB=NULL;
e=rnorm(nD)
sm=sample(1:m, 3, replace = FALSE)
i=sm[1]; j=sm[2]; k=sm[3]; print(c(i,j,k));
randsampleORIG=rbind(randsampleORIG, c(i, j, k));
randsample=rbind(randsample, sort(sm))
y=35+3*X[,sm[1]] +5*X[,sm[2]]+ X[,sm[3]] +e
BB=cbind(y,Dpop, X, rep(sm[1], n), rep(sm[2], n),  rep(sm[3], n))
AA=rbind(AA,BB);
```

```
}
print(randsample);
seed110=randsample;print(seed110);

set.seed(120);
for (i in 1:10)
{
BB=NULL;
e=rnorm(nD)
sm=sample(1:m, 3, replace = FALSE)
i=sm[1]; j=sm[2]; k=sm[3]; print(c(i,j,k));
randsampleORIG=rbind(randsampleORIG, c(i, j, k));
randsample=rbind(randsample, sort(sm))
y=35+3*X[,sm[1]] +5*X[,sm[2]]+ X[,sm[3]] +e
BB=cbind(y,Dpop, X, rep(sm[1], n), rep(sm[2], n),  rep(sm[3], n))
AA=rbind(AA,BB);
}
print(randsample);
seed120=randsample;print(seed120);

set.seed(130);
for (i in 1:10)
{
BB=NULL;
e=rnorm(nD)
```

```
sm=sample(1:m, 3, replace = FALSE)

i=sm[1]; j=sm[2]; k=sm[3]; print(c(i,j,k));

randsampleORIG=rbind(randsampleORIG, c(i, j, k));

randsample=rbind(randsample, sort(sm))

y=35+3*X[,sm[1]] +5*X[,sm[2]]+ X[,sm[3]] +e

BB=cbind(y,Dpop, X, rep(sm[1], n), rep(sm[2], n),  rep(sm[3], n))

AA=rbind(AA,BB);

}

print(randsample);

print(randsampleORIG);


seed130=randsample;print(seed130);

write.table(AA, "H:/Stapleton-Project/simulation_drought.csv",sep = ",")




##~~~~~~~~~Second 50 Replica ~~~~~~~~~~~~~~~~##

randsample=NULL; randsampleORIG=NULL; AA=NULL;


set.seed(140);

for (i in 1:10)

{

BB=NULL;

e=rnorm(nD)

sm=sample(1:m, 3, replace = FALSE)
```

```
i=sm[1]; j=sm[2]; k=sm[3]; print(c(i,j,k));
randsampleORIG=rbind(randsampleORIG, c(i, j, k));
randsample=rbind(randsample, sort(sm))
y=35+3*X[,sm[1]] +5*X[,sm[2]]+ X[,sm[3]] +e
BB=cbind(y,Dpop, X, rep(sm[1], n), rep(sm[2], n),  rep(sm[3], n))
AA=rbind(AA,BB);
}
print(randsample);
seed140=randsample;print(seed140);


set.seed(150);
for (i in 1:10)
{
BB=NULL;
e=rnorm(nD)
sm=sample(1:m, 3, replace = FALSE)
i=sm[1]; j=sm[2]; k=sm[3]; print(c(i,j,k));
randsampleORIG=rbind(randsampleORIG, c(i, j, k));
randsample=rbind(randsample, sort(sm))
y=35+3*X[,sm[1]] +5*X[,sm[2]]+ X[,sm[3]] +e
BB=cbind(y,Dpop, X, rep(sm[1], n), rep(sm[2], n),  rep(sm[3], n))
AA=rbind(AA,BB);
}
print(randsample);
seed150=randsample;print(seed150);
```

```
set.seed(160);
for (i in 1:10)
{
BB=NULL;
e=rnorm(nD)
sm=sample(1:m, 3, replace = FALSE)
i=sm[1]; j=sm[2]; k=sm[3]; print(c(i,j,k));
randsampleORIG=rbind(randsampleORIG, c(i, j, k));
randsample=rbind(randsample, sort(sm))
y=35+3*X[,sm[1]] +5*X[,sm[2]]+ X[,sm[3]] +e
BB=cbind(y,Dpop, X, rep(sm[1], n), rep(sm[2], n),  rep(sm[3], n))
AA=rbind(AA,BB);
}
print(randsample);
seed160=randsample;print(seed160);

set.seed(170);
for (i in 1:10)
{
BB=NULL;
e=rnorm(nD)
sm=sample(1:m, 3, replace = FALSE)
i=sm[1]; j=sm[2]; k=sm[3]; print(c(i,j,k));
randsampleORIG=rbind(randsampleORIG, c(i, j, k));
```

```
randsample=rbind(randsample, sort(sm))
y=35+3*X[,sm[1]] +5*X[,sm[2]]+ X[,sm[3]] +e
BB=cbind(y,Dpop, X, rep(sm[1], n), rep(sm[2], n),  rep(sm[3], n))
AA=rbind(AA,BB);
}
print(randsample);
seed170=randsample;print(seed170);

set.seed(180);
for (i in 1:10)
{
BB=NULL;
e=rnorm(nD)
sm=sample(1:m, 3, replace = FALSE)
i=sm[1]; j=sm[2]; k=sm[3]; print(c(i,j,k));
randsampleORIG=rbind(randsampleORIG, c(i, j, k));
randsample=rbind(randsample, sort(sm))
y=35+3*X[,sm[1]] +5*X[,sm[2]]+ X[,sm[3]] +e
BB=cbind(y,Dpop, X, rep(sm[1], n), rep(sm[2], n),  rep(sm[3], n))
AA=rbind(AA,BB);
}
print(randsample);
print(randsampleORIG);

seed180=randsample;print(seed180);
```

```
write.table(AA, "H:/Stapleton-Project/simulation_drought.csv",sep = ",")



##~~~~~~~~~Third 50 Replica ~~~~~~~~~~~~~~~~~##
randsample=NULL; randsampleORIG=NULL; AA=NULL;

set.seed(1234);
for (i in 1:50)
{
BB=NULL;
e=rnorm(nD)
sm=sample(1:m, 3, replace = FALSE)
i=sm[1]; j=sm[2]; k=sm[3]; print(c(i,j,k));
randsampleORIG=rbind(randsampleORIG, c(i, j, k));
randsample=rbind(randsample, sort(sm))
y=35+3*X[,sm[1]] +5*X[,sm[2]]+ X[,sm[3]] +e
BB=cbind(y,Dpop, X, rep(sm[1], n), rep(sm[2], n),  rep(sm[3], n))
AA=rbind(AA,BB);
}
print(randsample);
print(randsampleORIG);

seed1234=randsample;print(seed1234);
write.table(AA, "H:/Stapleton-Project/simulation_drought.csv",sep = ",")
```

```
##~~~~~~~~~Fourth 50 Replica ~~~~~~~~~~~~~~~~~##
randsample=NULL; randsampleORIG=NULL; AA=NULL;

set.seed(5678);
for (i in 1:50)
{
BB=NULL;
e=rnorm(nD)
sm=sample(1:m, 3, replace = FALSE)
i=sm[1]; j=sm[2]; k=sm[3]; print(c(i,j,k));
randsampleORIG=rbind(randsampleORIG, c(i, j, k));
randsample=rbind(randsample, sort(sm))
y=35+3*X[,sm[1]] +5*X[,sm[2]]+ X[,sm[3]] +e
BB=cbind(y,Dpop, X, rep(sm[1], n), rep(sm[2], n),  rep(sm[3], n))
AA=rbind(AA,BB);
}
print(randsample);
print(randsampleORIG);

seed5678=randsample;print(seed5678);
write.table(AA, "H:/Stapleton-Project/simulation_drought.csv",sep = ",")
```

##~~~~~~~~~ Totally 200 Replica above ~~~~~~~~~~~~~~~~##

#seeds=rbind(seed200, seed100, seed110, seed120, seed130, seed140, seed150, seed160, seed170, seed180,seed1234, seed5678)

#seeds100=rbind(seed200, seed100, seed110, seed120, seed130, seed140, seed150, seed160, seed170, seed180)

#seeds200=rbind(seed1234, seed5678)

#write.table(seeds, "H:/Stapleton-Project/seeds.csv",sep = ",")

#write.table(seeds100, "H:/Stapleton-Project/seeds100.csv",sep = ",")

#write.table(seeds200, "H:/Stapleton-Project/seeds200.csv",sep = ",")

#write.table(AA, "H:/Stapleton-Project/simulation_drought.csv",sep = ",")

SAS code for simulations

```
option nodate;
libname GENOANA 'H:\Stapleton-Project';


/***********************************************/
/*       Use GLMSELECT to build a model.       */
/*       First create a macro to hold the      */
/*       pop and marker*pop terms.             */
/***********************************************/
%let factor = pop;

%macro makefactor;
      %do i = 1 %to 1106;
            %let factor = &factor pop*m&i;
      %end;
%mend;
%makefactor;
%put &factor;                    /*  checks that factor is correct   */
```

```
PROC IMPORT OUT= GENOANA.simud
            DATAFILE= "H:\Stapleton-Project\simulation_drought.csv"
            DBMS=CSV REPLACE;
     GETNAMES=YES;
     DATAROW=2;
RUN;


data GENOANA.SIMUD(rename=(y=order Dpop=y VAR3=pop var4-var1109=m1-m1106 ));
set GENOANA.SIMUD;
*proc print;
run;


/*******************************************************************/
/*  Run glmselect.                                                 */
/*  The correct significance level to use depends on the markers   */
/*  and should be determined using a permutation analysis.         */
/*  The stop parameter may be set to a higher or lower value       */
/*  to limit the amount of time taken by the analysis.             */
/*******************************************************************/
/*
%macro select;
%do i = 1 %to 2;
data GENOANA.SIMUD;

%let n1=717*(&i-1)+1;
%let n2=717*&i;
set GENOANA.SIMUD (firstobs=n1 obs=n2);
run;

proc glmselect data=GENOANA.SIMUD;
class pop;
model y = &factor /select=sl sle=1e-2 sls=1e-2 stop=50 ;
run;
%end;
%mend;
%select;
*/


/*
%macro LSMeansAnalysis;
%do i=1 %to &_GLSNUMBYS;
title1 "Analysis Using the Selected Model for BY group number &i";
title2 "Selected Effects: &&_GLSIND&i";
ods select LSMeans;
proc glm data=glmselectOutput(where = (_BY_ = &i));
class classVar;
model y = &&_GLSIND&i;
lsmeans classVar;
run;quit;
%end;
%mend;
%LSMeansAnalysis;*/
```

```
/*~~~~~~~~~~ 1-10 ~~~~~~~~~~~~~~~~~~*/
title "G1";
data GENOANA.SIMUD1;
set GENOANA.SIMUD (firstobs=1 obs=717);
run;
proc glmselect data=GENOANA.SIMUD1 ;
class pop;
model y = &factor /select=sl sle=1e-2 sls=1e-2 stop=50 /*details=all
stats=all*/;
run;


title "G2";
data GENOANA.SIMUD2;
set GENOANA.SIMUD (firstobs=718 obs=1434);
run;
proc glmselect data=GENOANA.SIMUD2 ;
class pop;
model y = &factor /select=sl sle=1e-2 sls=1e-2 stop=50 /*details=all
stats=all*/;
run;


title "G3";
data GENOANA.SIMUD3;
set GENOANA.SIMUD (firstobs=1435 obs=2151);
run;
proc glmselect data=GENOANA.SIMUD3 ;
class pop;
model y = &factor /select=sl sle=1e-2 sls=1e-2 stop=50 /*details=all
stats=all*/;
run;


title "G4";
data GENOANA.SIMUD4;
set GENOANA.SIMUD (firstobs=2152 obs=2868);
run;
proc glmselect data=GENOANA.SIMUD4 ;
class pop;
model y = &factor /select=sl sle=1e-2 sls=1e-2 stop=50 /*details=all
stats=all*/;
run;


title "G5";
data GENOANA.SIMUD5;
set GENOANA.SIMUD (firstobs=2869 obs=3585);
run;
proc glmselect data=GENOANA.SIMUD5 ;
class pop;
```

```
model y = &factor /select=sl sle=1e-2 sls=1e-2 stop=50 /*details=all
stats=all*/;
run;



title "G6";
data GENOANA.SIMUD6;
set GENOANA.SIMUD (firstobs=3586 obs=4302);
run;
proc glmselect data=GENOANA.SIMUD6 ;
class pop;
model y = &factor /select=sl sle=1e-2 sls=1e-2 stop=50 /*details=all
stats=all*/;
run;


title "G7";
data GENOANA.SIMUD7;
set GENOANA.SIMUD (firstobs=4303 obs=5019);
run;
proc glmselect data=GENOANA.SIMUD7 ;
class pop;
model y = &factor /select=sl sle=1e-2 sls=1e-2 stop=50 /*details=all
stats=all*/;
run;


title "G8";
data GENOANA.SIMUD8;
set GENOANA.SIMUD (firstobs=5020 obs=5736);
run;
proc glmselect data=GENOANA.SIMUD8 ;
class pop;
model y = &factor /select=sl sle=1e-2 sls=1e-2 stop=50 /*details=all
stats=all*/;
run;



title "G9";
data GENOANA.SIMUD9;
set GENOANA.SIMUD (firstobs=5737 obs=6453);
run;
proc glmselect data=GENOANA.SIMUD9 ;
class pop;
model y = &factor /select=sl sle=1e-2 sls=1e-2 stop=50 /*details=all
stats=all*/;
run;


title "G10";
data GENOANA.SIMUD10;
set GENOANA.SIMUD (firstobs=6454 obs=7170);
run;
proc glmselect data=GENOANA.SIMUD10 ;
class pop;
```

```
model y = &factor /select=sl sle=1e-2 sls=1e-2 stop=50 /*details=all
stats=all*/;
run;



/*~~~~~~~~~ 11-20 ~~~~~~~~~~~~~~~~~~*/
title "G11";
data GENOANA.SIMUD11;
set GENOANA.SIMUD (firstobs=7171 obs=7887);
run;
proc glmselect data=GENOANA.SIMUD11 ;
class pop;
model y = &factor /select=sl sle=1e-2 sls=1e-2 stop=50 /*details=all
stats=all*/;
run;

title "G12";
data GENOANA.SIMUD12;
set GENOANA.SIMUD (firstobs=7888 obs=8604);
run;
proc glmselect data=GENOANA.SIMUD12 ;
class pop;
model y = &factor /select=sl sle=1e-2 sls=1e-2 stop=50 /*details=all
stats=all*/;
run;

title "G13";
data GENOANA.SIMUD13;
set GENOANA.SIMUD (firstobs=8605 obs=9321);
run;
proc glmselect data=GENOANA.SIMUD13 ;
class pop;
model y = &factor /select=sl sle=1e-2 sls=1e-2 stop=50 /*details=all
stats=all*/;
run;

title "G14";
data GENOANA.SIMUD14;
set GENOANA.SIMUD (firstobs=9322 obs=10038);
run;
proc glmselect data=GENOANA.SIMUD14 ;
class pop;
model y = &factor /select=sl sle=1e-2 sls=1e-2 stop=50 /*details=all
stats=all*/;
run;

title "G15";
data GENOANA.SIMUD15;
set GENOANA.SIMUD (firstobs=10039  obs=10755);
run;
proc glmselect data=GENOANA.SIMUD15 ;
class pop;
model y = &factor /select=sl sle=1e-2 sls=1e-2 stop=50 /*details=all
stats=all*/;
run;
```

```
 title "G16";
data GENOANA.SIMUD16;
set GENOANA.SIMUD (firstobs=10756   obs=11472);
run;
proc glmselect data=GENOANA.SIMUD16 ;
class pop;
model y = &factor /select=sl sle=1e-2 sls=1e-2 stop=50 /*details=all
stats=all*/;
run;

title "G17";
data GENOANA.SIMUD17;
set GENOANA.SIMUD (firstobs=11473   obs=12189 );
run;
proc glmselect data=GENOANA.SIMUD17 ;
class pop;
model y = &factor /select=sl sle=1e-2 sls=1e-2 stop=50 /*details=all
stats=all*/;
run;

title "G18";
data GENOANA.SIMUD18;
set GENOANA.SIMUD (firstobs=12190    obs=12906 );
run;
proc glmselect data=GENOANA.SIMUD18 ;
class pop;
model y = &factor /select=sl sle=1e-2 sls=1e-2 stop=50 /*details=all
stats=all*/;
run;


title "G19";
data GENOANA.SIMUD19;
set GENOANA.SIMUD (firstobs=12907     obs=13623 );
run;
proc glmselect data=GENOANA.SIMUD19 ;
class pop;
model y = &factor /select=sl sle=1e-2 sls=1e-2 stop=50 /*details=all
stats=all*/;
run;


title "G20";
data GENOANA.SIMUD20;
set GENOANA.SIMUD (firstobs=13624  obs=14340);
run;
proc glmselect data=GENOANA.SIMUD20 ;
class pop;
model y = &factor /select=sl sle=1e-2 sls=1e-2 stop=50 /*details=all
stats=all*/;
run;



/*~~~~~~~~~~ 21-30 ~~~~~~~~~~~~~~~~~~*/
title "G21";
data GENOANA.SIMUD21;
```

```
set GENOANA.SIMUD (firstobs=14341 obs=15057);
run;
proc glmselect data=GENOANA.SIMUD21 ;
class pop;
model y = &factor /select=sl sle=1e-2 sls=1e-2 stop=50 /*details=all
stats=all*/;
run;



title "G22";
data GENOANA.SIMUD22;
set GENOANA.SIMUD (firstobs=15058 obs=15774);
run;
proc glmselect data=GENOANA.SIMUD22;
class pop;
model y = &factor /select=sl sle=1e-2 sls=1e-2 stop=50 /*details=all
stats=all*/;
run;

title "G23";
data GENOANA.SIMUD23;
set GENOANA.SIMUD (firstobs=15775 obs=16491);
run;
proc glmselect data=GENOANA.SIMUD23;
class pop;
model y = &factor /select=sl sle=1e-2 sls=1e-2 stop=50 /*details=all
stats=all*/;
run;

title "G24";
data GENOANA.SIMUD24;
set GENOANA.SIMUD (firstobs=16492 obs=17208);
run;
proc glmselect data=GENOANA.SIMUD24;
class pop;
model y = &factor /select=sl sle=1e-2 sls=1e-2 stop=50 /*details=all
stats=all*/;
run;


title "G25";
data GENOANA.SIMUD25;
set GENOANA.SIMUD (firstobs=17209 obs=17925);
run;
proc glmselect data=GENOANA.SIMUD25;
class pop;
model y = &factor /select=sl sle=1e-2 sls=1e-2 stop=50 /*details=all
stats=all*/;
run;


title "G26";
data GENOANA.SIMUD26;
set GENOANA.SIMUD (firstobs=17926 obs=18642);
run;
proc glmselect data=GENOANA.SIMUD26;
```

```
class pop;
model y = &factor /select=sl sle=1e-2 sls=1e-2 stop=50 /*details=all
stats=all*/;
run;

title "G27";
data GENOANA.SIMUD27;
set GENOANA.SIMUD (firstobs=18643 obs=19359 );
run;
proc glmselect data=GENOANA.SIMUD27;
class pop;
model y = &factor /select=sl sle=1e-2 sls=1e-2 stop=50 /*details=all
stats=all*/;
run;

title "G28";
data GENOANA.SIMUD28;
set GENOANA.SIMUD (firstobs= 19360 obs=20076);
run;
proc glmselect data=GENOANA.SIMUD28;
class pop;
model y = &factor /select=sl sle=1e-2 sls=1e-2 stop=50 /*details=all
stats=all*/;
run;

title "G29";
data GENOANA.SIMUD29;
set GENOANA.SIMUD (firstobs= 20077 obs=20793);
run;
proc glmselect data=GENOANA.SIMUD29;
class pop;
model y = &factor /select=sl sle=1e-2 sls=1e-2 stop=50 /*details=all
stats=all*/;
run;



title "G30";
data GENOANA.SIMUD30;
set GENOANA.SIMUD (firstobs= 20794 obs=21510);
run;
proc glmselect data=GENOANA.SIMUD30;
class pop;
model y = &factor /select=sl sle=1e-2 sls=1e-2 stop=50 /*details=all
stats=all*/;
run;


/*~~~~~~~~~ 31-40 ~~~~~~~~~~~~~~~~~*/
title "G31";
data GENOANA.SIMUD31;
set GENOANA.SIMUD (firstobs= 21511 obs=22227);
run;
proc glmselect data=GENOANA.SIMUD31;
class pop;
model y = &factor /select=sl sle=1e-2 sls=1e-2 stop=50 /*details=all
stats=all*/;
```

```
run;

title "G32";
data GENOANA.SIMUD32;
set GENOANA.SIMUD (firstobs= 22228 obs=22944);
run;
proc glmselect data=GENOANA.SIMUD32;
class pop;
model y = &factor /select=sl sle=1e-2 sls=1e-2 stop=50 /*details=all
stats=all*/;
run;

title "G33";
data GENOANA.SIMUD33;
set GENOANA.SIMUD (firstobs= 22945 obs=23661);
run;
proc glmselect data=GENOANA.SIMUD33;
class pop;
model y = &factor /select=sl sle=1e-2 sls=1e-2 stop=50 /*details=all
stats=all*/;
run;

title "G34";
data GENOANA.SIMUD34;
set GENOANA.SIMUD (firstobs= 23662 obs=24378);
run;
proc glmselect data=GENOANA.SIMUD34;
class pop;
model y = &factor /select=sl sle=1e-2 sls=1e-2 stop=50 /*details=all
stats=all*/;
run;

title "G35";
data GENOANA.SIMUD35;
set GENOANA.SIMUD (firstobs= 24379 obs=25095);
run;
proc glmselect data=GENOANA.SIMUD35;
class pop;
model y = &factor /select=sl sle=1e-2 sls=1e-2 stop=50 /*details=all
stats=all*/;
run;

title "G36";
data GENOANA.SIMUD36;
set GENOANA.SIMUD (firstobs= 25096 obs=25812);
run;
proc glmselect data=GENOANA.SIMUD36;
class pop;
model y = &factor /select=sl sle=1e-2 sls=1e-2 stop=50 /*details=all
stats=all*/;
run;

title "G37";
data GENOANA.SIMUD37;
set GENOANA.SIMUD (firstobs= 25813 obs=26529);
run;
proc glmselect data=GENOANA.SIMUD37;
```

```
class pop;
model y = &factor /select=sl sle=1e-2 sls=1e-2 stop=50 /*details=all
stats=all*/;
run;

title "G38";
data GENOANA.SIMUD38;
set GENOANA.SIMUD (firstobs= 26530 obs=27246);
run;
proc glmselect data=GENOANA.SIMUD38;
class pop;
model y = &factor /select=sl sle=1e-2 sls=1e-2 stop=50 /*details=all
stats=all*/;
run;

title "G39";
data GENOANA.SIMUD39;
set GENOANA.SIMUD (firstobs= 27247 obs=27963);
run;
proc glmselect data=GENOANA.SIMUD39;
class pop;
model y = &factor /select=sl sle=1e-2 sls=1e-2 stop=50 /*details=all
stats=all*/;
run;

title "G40";
data GENOANA.SIMUD40;
set GENOANA.SIMUD (firstobs= 27964 obs=28680);
run;
proc glmselect data=GENOANA.SIMUD40;
class pop;
model y = &factor /select=sl sle=1e-2 sls=1e-2 stop=50 /*details=all
stats=all*/;
run;



/*~~~~~~~~~~ 41-50 ~~~~~~~~~~~~~~~~~~*/
title "G41";
data GENOANA.SIMUD41;
set GENOANA.SIMUD (firstobs= 28681 obs=29397);
run;
proc glmselect data=GENOANA.SIMUD41;
class pop;
model y = &factor /select=sl sle=1e-2 sls=1e-2 stop=50 /*details=all
stats=all*/;
run;

title "G42";
data GENOANA.SIMUD42;
set GENOANA.SIMUD (firstobs= 29398 obs=30114);
run;
proc glmselect data=GENOANA.SIMUD42;
class pop;
model y = &factor /select=sl sle=1e-2 sls=1e-2 stop=50 /*details=all
stats=all*/;
run;
```

```
title "G43";
data GENOANA.SIMUD43;
set GENOANA.SIMUD (firstobs= 30115 obs=30831);
run;
proc glmselect data=GENOANA.SIMUD43;
class pop;
model y = &factor /select=sl sle=1e-2 sls=1e-2 stop=50 /*details=all
stats=all*/;
run;

title "G44";
data GENOANA.SIMUD44;
set GENOANA.SIMUD (firstobs= 30832 obs=31548);
run;
proc glmselect data=GENOANA.SIMUD44;
class pop;
model y = &factor /select=sl sle=1e-2 sls=1e-2 stop=50 /*details=all
stats=all*/;
run;


title "G45";
data GENOANA.SIMUD45;
set GENOANA.SIMUD (firstobs= 31549 obs=32265);
run;
proc glmselect data=GENOANA.SIMUD45;
class pop;
model y = &factor /select=sl sle=1e-2 sls=1e-2 stop=50 /*details=all
stats=all*/;
run;

title "G46";
data GENOANA.SIMUD46;
set GENOANA.SIMUD (firstobs= 32266 obs=32982);
run;
proc glmselect data=GENOANA.SIMUD46;
class pop;
model y = &factor /select=sl sle=1e-2 sls=1e-2 stop=50 /*details=all
stats=all*/;
run;

title "G47";
data GENOANA.SIMUD47;
set GENOANA.SIMUD (firstobs= 32983 obs=33699);
run;
proc glmselect data=GENOANA.SIMUD47;
class pop;
model y = &factor /select=sl sle=1e-2 sls=1e-2 stop=50 /*details=all
stats=all*/;
run;

title "G48";
data GENOANA.SIMUD48;
set GENOANA.SIMUD (firstobs= 33700 obs=34416);
run;
```

```
proc glmselect data=GENOANA.SIMUD48;
class pop;
model y = &factor /select=sl sle=1e-2 sls=1e-2 stop=50 /*details=all
stats=all*/;
run;

title "G49";
data GENOANA.SIMUD49;
set GENOANA.SIMUD (firstobs= 34417 obs=35133);
run;
proc glmselect data=GENOANA.SIMUD49;
class pop;
model y = &factor /select=sl sle=1e-2 sls=1e-2 stop=50 /*details=all
stats=all*/;
run;


title "G50";
data GENOANA.SIMUD50;
set GENOANA.SIMUD (firstobs= 35134 obs=35850);
run;
proc glmselect data=GENOANA.SIMUD50;
class pop;
model y = &factor /select=sl sle=1e-2 sls=1e-2 stop=50 /*details=all
stats=all*/;
run;
```
