# Supplementary material for: Genotype to Phenotype Maps: Multiple Input Abiotic Signals Combine to Produce Growth Effects via Attenuating Signaling Interactions in Maize
Source: G3 (Bethesda). 2013 Oct 18;3(12):2195–204. doi: 10.1534/g3.113.008573 (PMC3852382; doi:10.1534/g3.113.008573)
Supplement: Supporting Information [file supp_g3.113.008573_TableS1.pdf]

Table S1 Positive predictive value of SLE thresholds for NAM marker selection. Output of R and SAS simulations, with positive predictive value calculated at true positives divided by true plus false positives.

Simulated QTL detected	SLE Threshold Tested		
	**P=0.002**	**P=0.001**	**P=0.005**
**True positives**	597	592	287
**False positives**	97	59	74
**Positive predictive value**	0.86	0.91	0.79

The highest positive predictive value was found for the SLE of 0.001; that value was used for analysis of the NAM data sets.
